# Supplementary material for: Identification of a Sgo2-Dependent but Mad2-Independent Pathway Controlling Anaphase Onset in Fission Yeast
Source: Cell Rep. 2017 Feb 7;18(6):1422–33. doi: 10.1016/j.celrep.2017.01.032 (PMC5316559; doi:10.1016/j.celrep.2017.01.032)
Supplement: Document S1. Supplemental Experimental Procedures, Figures S1–S5, and Table S2 [file mmc1.pdf]

Cell Reports, Volume *18*

Supplemental Information

Identification of a Sgo2-Dependent but Mad2-Independent Pathway Controlling Anaphase Onset in Fission Yeast

John C. Meadows, Theresa C. Lancaster, Graham J. Buttrick, Alicja M. Sochaj, Liam J. Messin, Maria del Mar Mora-Santos, Kevin G. Hardwick, and Jonathan B.A. Millar

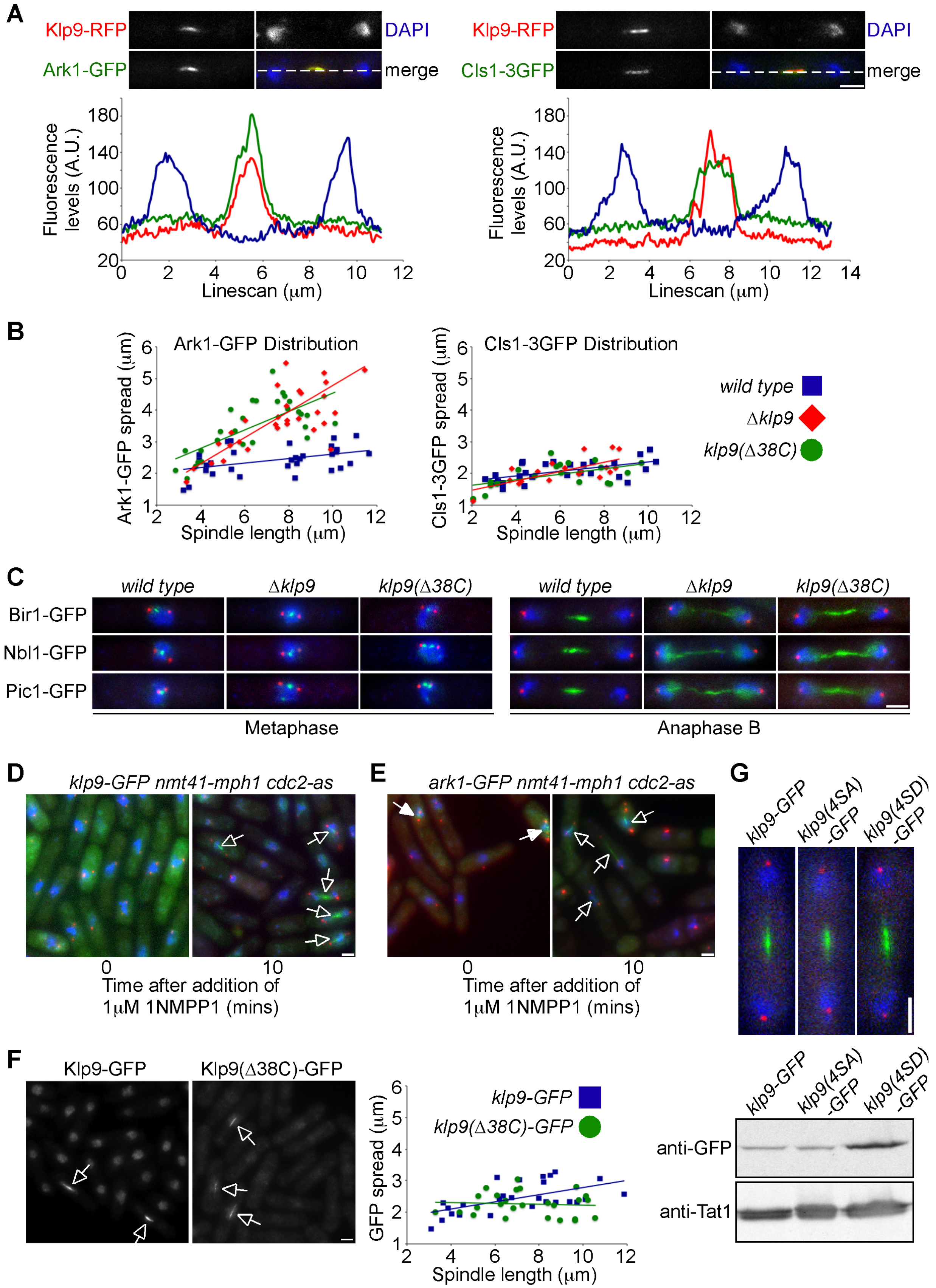

Figure S1, relating to Figure 1

Figure S1, relating to Figure 1. CPC component re-localisation requires Klp9 C-terminus and is promoted by Cdk1 inactivity

(A) Klp9 co-localises with Ark1 and Cls1 on the spindle midzone in anaphase B. Representative cells (top row panels) expressing Klp9-RFP (red) and either Ark1-GFP or Cls1-3GFP (green) with chromatin stained with DAPI (blue). All bars, 2μm. The dashed white line represents a 1 pixel wide linescan with relative fluorescence levels graphed directly below.
(B) Scatter plots to quantify Ark1-GFP and Cls1-3GFP distribution against pole-to-pole spindle length in the presence of either full length ("*wild type*"), truncated ("*klp9(Δ38C)*") or absent ("Δ*klp9*") Klp9 as shown in Figures 1B & 1G.
(C) Re-localisation of CPC components Bir1, Nbl1 & Pic1 is disrupted in the absence of the C-terminus of Klp9. Representative images of cells expressing either Bir1-GFP, Nbl1-GFP or Pic1-GFP (all green), Sid4-TdTomato (SPBs, red) and chromatin stained with DAPI (blue) in metaphase and anaphase B in either the absence of Klp9 ("Δ*klp9*"), or presence of C-terminally truncated ("*klp9(Δ38C)*") or full length Klp9 ("*wild type*").
(D) Inactivation of Cdk1 promotes localisation of Klp9 to the spindle midzone. Cells expressing Klp9-GFP (green), Sid4-TdTomato (SPBs, red), Cdc2-as (analogue-sensitive Cdc2) and a thiamine repressible promoter Mph1 construct (*nmt41-mph1*) were grown to mid-log phase in in the presence of thiamine and then transferred to medium lacking thiamine to overexpress Mph1 for 16 hours. Note lack of Klp9-GFP signal at time "0" (left panel). 10 minutes after inactivation of Cdc2 by addition of 1μM 1NMPP1, Klp9-GFP can be seen on spindles (arrows, right panel).
(E) Inactivation of Cdk1 promotes relocalisation of Ark1 to the spindle midzone. Experiment as for (D) but using cells expressing Ark1-GFP in place of Klp9-GFP. Metaphase-arrested cells (left panel, "0") have Ark1-GFP on centromeres (closed arrow-heads). Cells imaged 10 minutes after the addition of 1¨M of 1NMPP1 to inhibit Cdc2 phosphorylation (right panel, "10") show that Ark1-GFP now localises to spindles (open arrow-heads).
(F) C-terminally truncated Klp9 localises to the spindle midzone in anaphase B. Cells expressing Klp9-GFP or Klp9(Δ38C)-GFP (left panels) were grown to mid-log phase and fixed. Arrows indicate mitotic cells with GFP signals on anaphase cells. Graph shows quantification of Klp9-GFP versus Klp9(Δ38C)-GFP signals as a function of spindle length.
(G) Phospho-mimetic and non-phosphorylatable Klp9-GFP localisation and expression levels. Cells expressing Klp9-GFP, Klp9(4SA)-GFP and Klp9(4SD)-GFP (top panel) were grown to mid-log phase and fixed. Western blot (bottom panel) of mid-log phase *klp9-GFP*, *klp9(4SA)-GFP* and *klp9(4SD)-GFP* cell extracts probed with anti-GFP antibody. Levels of tubulin (anti-Tat1) are used as a loading control.

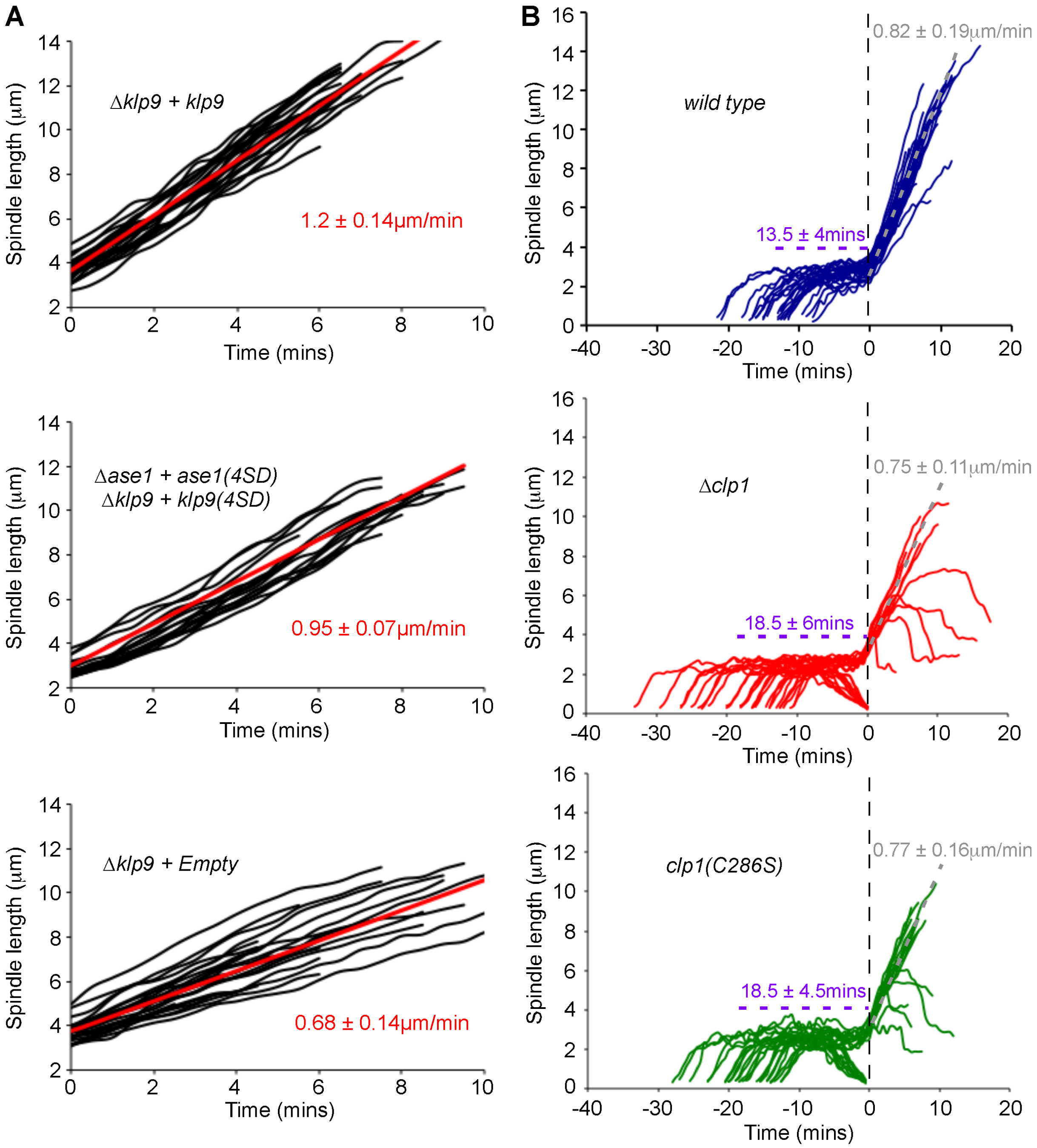

Figure S2, relating to Figure 1

Figure S2, relating to Figure 1. Neither dephosphorylation of the Klp9 C-terminus nor Cdc14-like phosphatase activity influence Anaphase B spindle elongation rate

(A) C-terminal phosphomimetic *ase1* and *klp9* alleles do not alter the rate of anaphase B spindle elongation. Log phase *sid4-TdTomato* cells deleted for *klp9* and with either *klp9 (Δklp9 + klp9*, top panel, n= 25) or empty plasmid (Δ*klp9* + *Empty*, bottom panel, n= 23) integrated or the same with *ase1* also deleted and expressing phosphomimetic *ase1* and *klp9* constructs (Δ*ase1* + *ase1(4SD)* Δ*klp9* + *klp9(4SD)*, middle panel, n= 17) were imaged by fluorescence microscopy at 30°C. Spindle length in individual anaphase B cells was calculated at 30 second intervals. Red lines indicate mean spindle elongation rates, which are quantified ± standard deviation.

(B) Neither the presence nor the activity of Cdc14-like phosphatase, Clp1, alter the rate of spindle elongation in anaphase B. Log phase *ndc80-GFP cdc11-CFP* cells (*wild type*, top panel, n = 29) or the same lacking Clp1 (Δ*clp1*, middle panel, n = 25) or expressing Clp1(C286S) (*clp1(C286S)*, bottom panel, n = 25) were imaged by fluorescence microscopy at 30°C. Spindle length in individual mitotic cells was calculated at 30 second intervals. The completion of anaphase A was taken as T = 0 for each movie. Spindle collapses are those traces in which spindle length reduces to zero. Average time in prometaphase & metaphase and anaphase B spindle elongation rate are shown ± standard deviation.

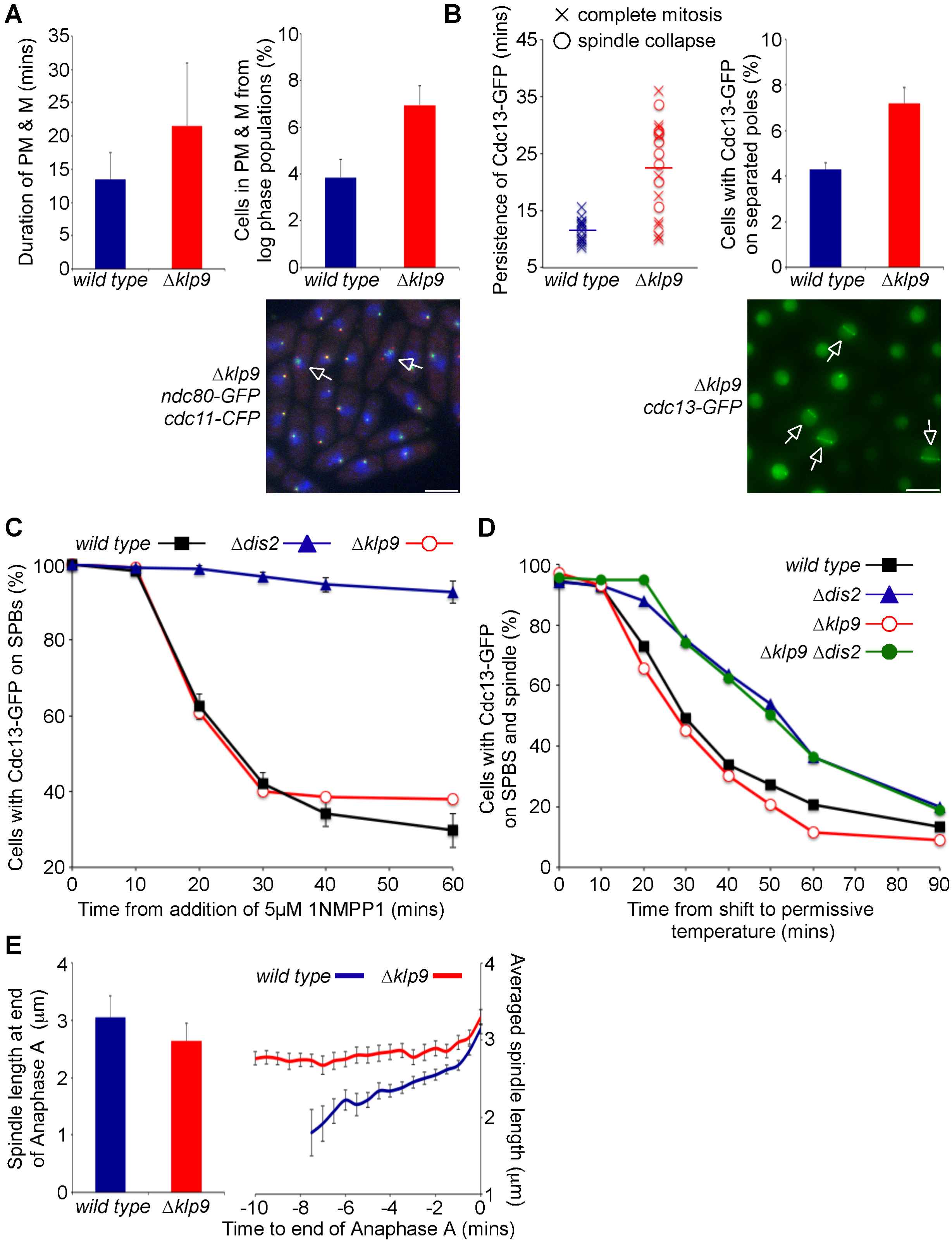

Figure S3, relating to Figure 2

Figure S3, relating to Figure 2. Klp9 is required for timely Cdc13 destruction due to prolonged SAC activation rather than defective silencing

(A) Comparison of the duration of prometaphase and metaphase (PM & M) from live cell analysis in Figure 2A with the proportion of PM & M cells in fixed images of mid-log phase cells either in the presence (*wild type*) or absence (Δ*klp9*) of Klp9. Error bars show standard deviation. Representative image (bottom panel) with PM & M cells highlighted with arrowheads. Spindle poles fluorescently-tagged with Cdc11-CFP (red) and kinetochores with Ndc80-GFP (green). All bars, 5µm.

(B) Cdc13 localisation persists in cells deleted for Klp9. Cells with and without Klp9 and expressing Cdc13-GFP were grown to mid-log phase at 30°C before conducting either live cell analysis (left panel) to determine the duration of GFP signal in individual mitotic cells or being fixed (right panel) and scored for GFP signal on separated poles. Horizontal lines show mean values (left panel) and error bars represent standard deviation (right panel). Representative image (bottom panel) with cells that localise Cdc13-GFP (green) on separated poles highlighted with arrowheads.

(C) Klp9 is not required for microtubule-independent spindle checkpoint silencing. Cells of the indicated genotypes expressing Cdc13-GFP (to monitor mitotic progression), Ark1-as3 (analogue-sensitive Aurora B to inactivate the spindle checkpoint) and Nda3-KM311 (cold-sensitive ß-tubulin mutant to arrest cells in a prometaphase-like state) were grown to mid-log phase at 30°C before being arrested for 6 hours at 18°C. 1NMPP1 was then added and samples collected at the indicated time points and assayed for the presence of Cdc13-GFP on poles.

(D) Klp9 does not influence the timing of anaphase onset following release from a spindle checkpoint arrest. Cdc13-GFP expressing cells containing the *nda3-KM311* allele were grown to mid-log phase at 30°C before being arrested in prometaphase-like state by shifting temperature to 18°C for 6 hours. Cells were then released at 30°C and the proportion of cells with Cdc13-GFP on poles and spindles assayed at the indicated time points.

(E) Spindle length at the end of anaphase A is not altered in the absence of Klp9. Left panel shows data extracted from time 0 in Figure 2A and the right panel shows data from the same cells that are analysed in Figure 2C.

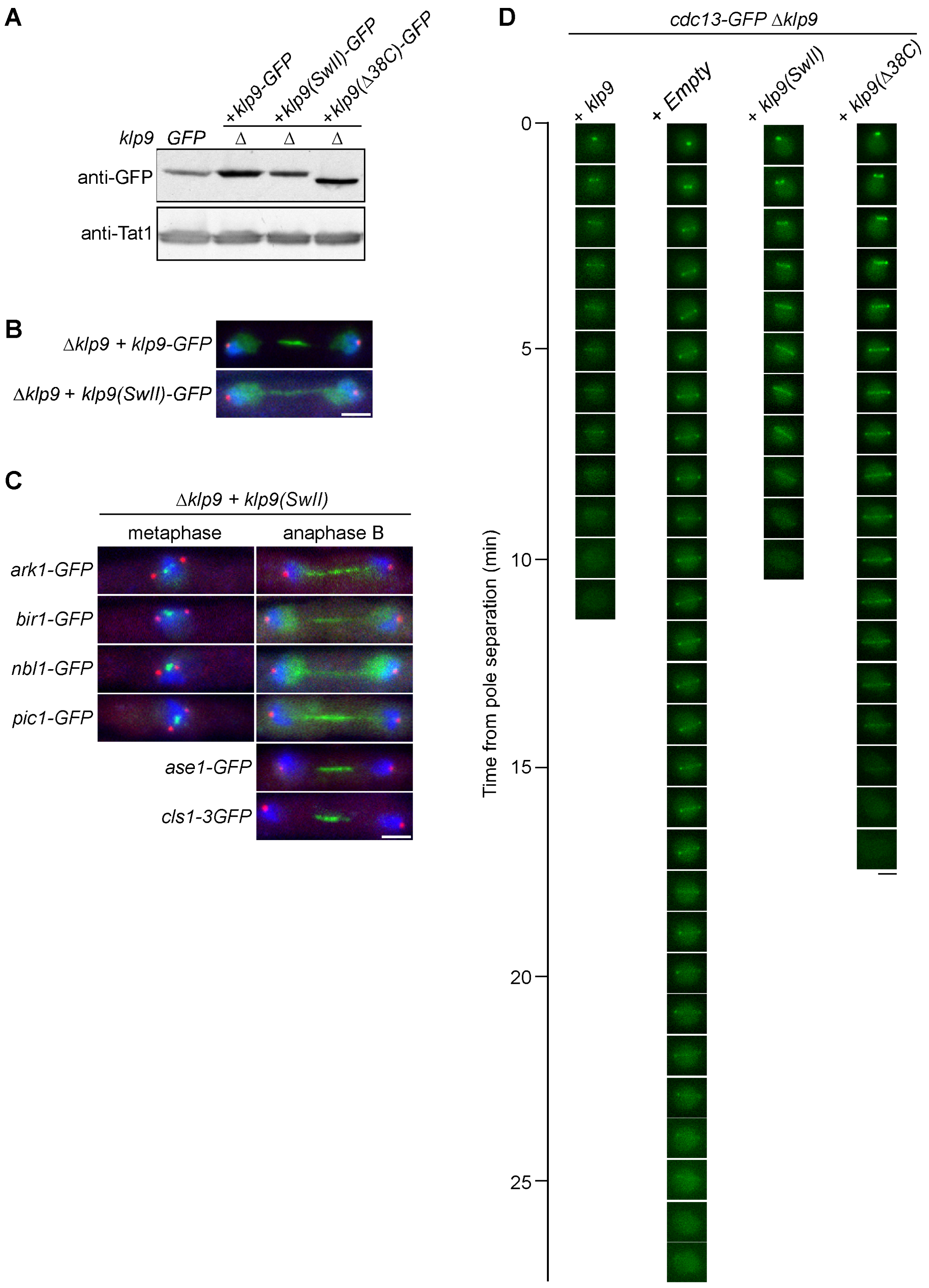

Figure S4, relating to Figure 3

Figure S4, relating to Figure 3. Klp9(SwII) characterisation

(A) Expression levels of *klp9:leu1* constructs. Mid-log phase *klp9-GFP, Δklp9 + klp9-GFP, Δklp9 + klp9(SwII)-GFP* and *klp9 + klp9(Δ38C)-GFP* cell extracts were probed with anti-GFP antibody. Levels of tubulin (anti-Tat1) are used as a loading control.
(B) Klp9-SwII does not localise to the spindle midzone. Δ*klp9* cells expressing either Klp9-GFP or Klp9(SwII)-GFP (green) form the *leu1* locus and Sid4-TdTomato (red) were grown to mid-log phase at 30°C and fixed. All bars, 2µm.
(C) CPC component relocalisation to the spindle midzone is disrupted in the absence of Klp9 motor activity. Δ*klp9 + klp9(SwII) sid4-TdTomato* cells expressing either Ark1-GFP, Bir1-GFP, Nbl1-GFP, Pic1-GFP, Ase1-GFP or Cls1-3GFP (green) were grown to mid-log phase at 30°C and fixed.
(D) Timely Cyclin B destruction requires the C-terminus of Klp9 but not its motor activity. Representative movies from the data quantified in Figure 3D.

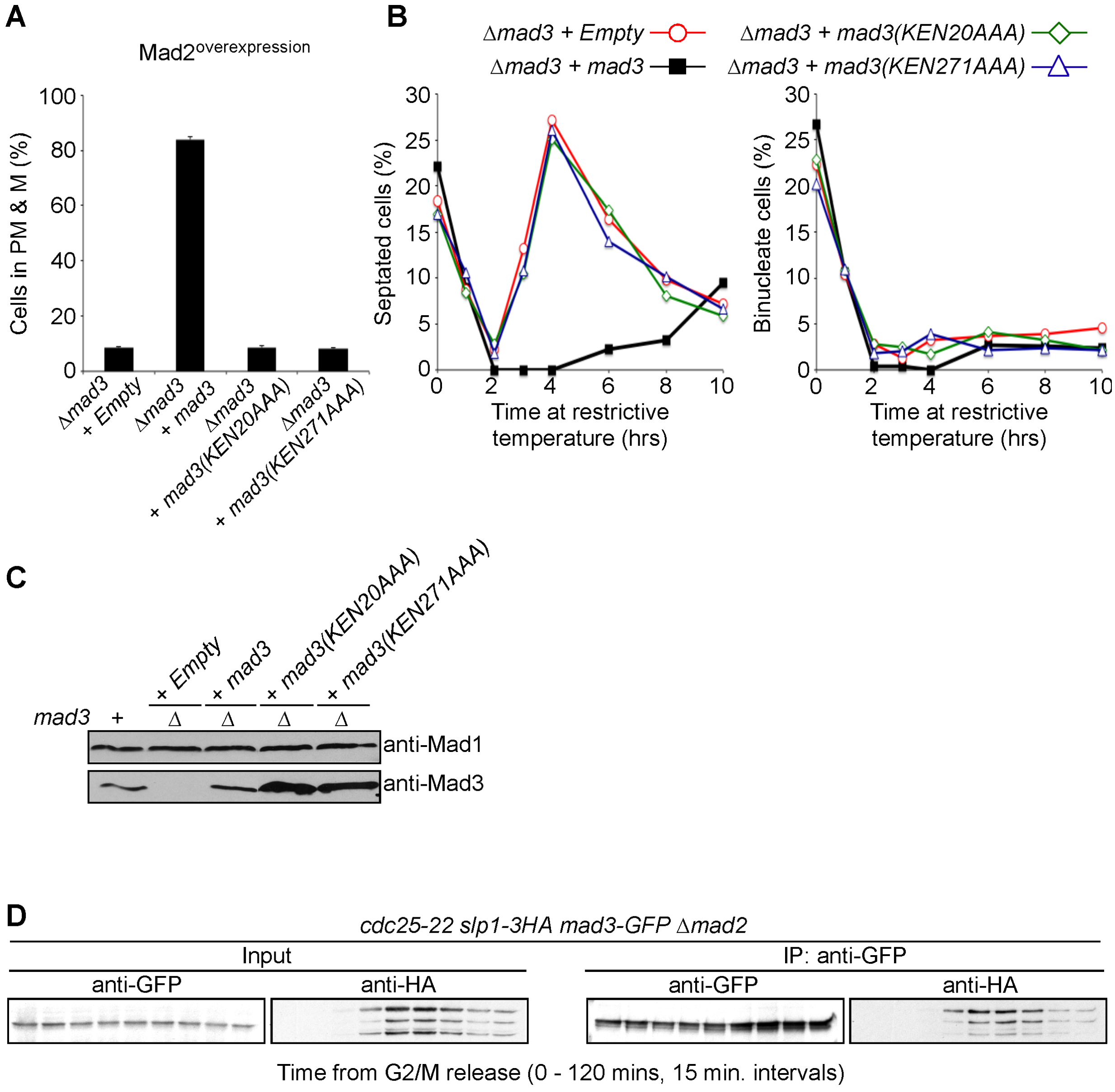

Figure S5, relating to Figure 5

Figure S5, relating to Figure 5. SAC dependency controls

(A) Mad3-KEN20AAA and Mad3-KEN271AAA are both defective in checkpoint arrest in response to over-expression of Mad2. *dad1-GFP sid4-TdTomato* cells deleted for Mad3 and expressing either empty plasmid (*Empty*), *mad3*, *mad3-KEN20AAA* or *mad3-KEN271AAA* were grown to mid-log phase at 30°C in media containing thiamine. They were then washed and resuspended in media lacking thiamine to allow overexpression of Mad2 under the control of a thiamine repressible promoter. After 16 hours cells were fixed and the proportion in Prometaphase & Metaphase (PM & M) assayed by fluorescence microscopy.

(B) Mad3-KEN20AAA and Mad3-KEN271AAA both fail to mount a checkpoint response to *nda3-KM311*-mediated checkpoint activation. Mid-log phase *nda3-KM311* cells with the genotypes detailed were shifted from 30°C to 18°C at time zero. Cells were sampled at the times indicated and stained with both calcofluor to assay the proportion of septated cells (left panel) and DAPI to stain chromatin and score the proportion of binucleate cells (right panel).

(C) Mad3 KEN box mutant proteins are present at wild type Mad3 levels. Lysates were prepared from log phase strains expressing either endogenous *mad3* or the various *mad3* constructs integrated at the *leu1* locus. Levels of Mad1 and Mad3 were assessed following western blotting.

(D) Mad3 and Slp1 interact in the absence of Mad2. *cdc25-22 slp1-3HA mad3-GFP* Δ*mad2* cells were arrested for 4 hours at 35.5˚C to synchronise at the G2/M transition. Following release at 25˚C, extracts were prepared every 15 minutes. Association between Slp1-3HA and Mad3-GFP was assessed by immunoprecipitation and western blot.

Table S1

List of fission yeast strains used in this study.

All strains are *ura4-D18 leu1-32*. *ade6* allele is unknown unless stated.

Table S2

List of oligonucleotides used in this study.

Bir1 tag.F	GAGTTTGAAGAAGCCTGTGAAGAAAAAATAGAATGGTTACTGGAAGAAGGTAAGCGGGCGGAAGAATACATACAAAACTTACGGATCCCCGGGTTAATTAA
Bir1 tag.Rev	CACATTACTTTAAAATGTAGCCCGCCAACATTTTCGCTTAAAGCGAGACGGTCCATAAATACAGAAACACAATATGTAAAAGAATTCGAGCTCGTTTAAAC
Klp9 del.F	AGCAACTGTCTTCCAACACTTCCATCATTCTTGTACACAAAGGATTGCTTTTAAGTCCCTTTAAACTCTGAAATTGACCACGGATCCCCGGGTTAATTAA
Klp9 tag.F	TAAAACCATTAAGCCCCTCTCGACGCCCTCCGTTGACTTCTTTGTATAGTGGGACAACTGATATCGATATTAATGAATTGCGGATCCCCGGGTTAATTAA
Klp9 del/tag.R	CATGTGAAGGCAAGAGCTAAATATTACTCAAATAAGAGTTATGAACACATTTTGCCATTCTTGGTAACTTTAATGATATAGAATTCGAGCTCGTTTAAAC
Klp9 XbaI.F	TGCTCTAGAGCAATCGCAGTC
Klp9 KpnI.R	TAAGGTACCAAATGCCTTCGTTTTAAA
Klp9 PstI.R	TTTCTGCAGTTAGGGTACGTTTTCCTCATCATC
Klp9 SwII.F	GATTTAGCCGCATCCGAGCGT
Klp9 SwII.R	CACCAAATCTATTTGACAACTTTCAAA
Klp9 S596A,S598A.F	GTACCCGCACCGGCTCCCAAAAAAAAAGTT
Klp9 S596D,S598D.F	GTACCCGATCCGGATCCCAAAAAAAAAGTT
Klp9 S596A or D.R	GTTTTCCTCATCATCACTATTTGG
Klp9 S598D,S605D.F	AGTCCGGATCCCAAAAAAAAAGTTGTAGATCCAATA
Klp9 S598D,S605D.R	GGGTACGTTTTCCTCATCATCACTATTTGG
Klp9 S605A,S611A.F	CCCAAAAAAAAAGTTGTAGCGCCAATAAAACAATTAGCGCCCTCTCGACGCCCTCCGTTG
Klp9 S605A,S611A.R	CAACGGAGGGCGTCGAGAGGGCGCTAATGGTTTTATTGGCGCTACAACTTTTTTTTTGGG
Klp9 S611D.F	CCATTAGATCCCTCTCGACGCCCT
Klp9 S611D.R	TTTTATTGGATCTACAACTTTTTTTTTGGG
Klp9(Δ38C).w	ACGATTGCGCAGTTAGAACAAATT
Klp9(Δ38C).x	GGGGATCCGTCGACCTGCAGCGTACGATTAGGGTACGTTTTCCTCATC
Klp9(Δ38C).y	GTTTAAACGAGCTCGAATTCATCGATATCCGTCGTGCATAGTGCTTA
Klp9(Δ38C).z	AGAGCTAAATATTACTCAAATAAGAGT
Klp9(Δ38C) tag.x	GGGGATCCGTCGACCTGCAGCGTACGAGGGTACGTTTTCCTCATC
Mad3 BamHI.F	CGCGGATCCTCTTGTAAATCGTTGTTTATAAATCTTCAT
Mad3 PstI.R	TTGGATCTGCAGGGAGTTGATTTTGTTTTGGTTCAA
Mad3 NruI(mut).F	AAATCTTCGTCCCGAAACCACA
Mad3 NruI(mut).R	TGCAAGTGCAGAAGCTGAATG
Mad3 KEN20.F	GTCATTGAGCAGTCCGCGGCAGCCATAGAGCCTAGAAAA
Mad3 KEN20.R	ATCCATATGCACCCAGTTCTTGCC
Mad3 KEN271.F	GTTGATCAAAGGCGTGCAGCAGCCAACATATCGGCTACT
Mad3 KEN271.R	TGTACCAAGAGTTTGCCAGGTCCC

Supplemental Experimental Procedures

Strain construction

Δ*klp9*, *klp9-GFP*, and *bir1-13myc* were generated using a single-step PCR-based protocol (Bahler et al., 1998). *klp9(Δ38C)* was produced by two-step PCR-based gene targeting as previously described (Krawchuk and Wahls, 1999). The resulting truncation was tagged with GFP using the same method. See Table S2 for a full list of oligonucleotides used in this study.

Plasmid construction

Klp9 was cloned as a 2.8kb fragment with 500bp of promoter region and 289bp of 3' UTR and inserted into the *XbaI* and *KpnI* sites of pJK148 and clones verified by sequencing. The Δ38C mutant was generated by a truncation at residue 595 and cloned into the *XbaI* and *PstI* sites of pJK148. Serines 596, 598, 605 and 611 were mutated to either alanine or aspartic acid and the Switch II (G296A) mutant generated by Phusion site-directed mutagenesis kit (Fisher Scientific) according to manufacturer's instructions.

Mad3 was cloned as a 1.8kb fragment containing 500bp of upstream promoter sequence and 440bp of 3' UTR and cloned into the *BamHI* and *PstI* sites of pJK148. To ensure the resulting plasmid was suitable for integration, the *NruI* restriction site at Serine 40 of *mad3* was mutated from TCG to TCC using Phusion Site-directed Mutagenesis kit and clones verified by sequencing. KEN box mutations were likewise generated using the Phusion Site-directed mutagenesis kit.

Plasmids were linearised with *NruI* and integrated into *S. pombe* strains with the *leu1.32* auxotrophic marker and confirmed by PCR (Keeney and Boeke, 1994). See Table S2 for a full list of oligonucleotides used in this study.

Biochemistry

Cells were lysed in buffer containing 50 mM HEPES (pH 7.6), 75 mM KCl, 1 mM $MgCl_2$, 1 mM EGTA, 0.1% Triton X-100, 1 mM DTT, 1 mM PMSF and c0mplete EDTA-free protease inhibitor cocktail (Roche). Cleared extracts were incubated with GFP-Trap_A beads (Chromotek) for 1 hour at 4°C. Beads were washed three times in lysis buffer and boiled in sample buffer.

GFP was detected using anti-GFP sheep polyclonal antibody (1/5000) and HRP conjugated anti-sheep secondary antibody (1/20000). Myc was detected using anti-Myc rabbit polyclonal antibody (1/500: A-14, Santa Cruz) and HRP conjugated anti-rabbit secondary antibody (1/10000: GE Healthcare). HA was detected using HRP-conjugated anti-HA rat monoclonal antibody (1/500: 3F10, Roche). Mad1 and Mad3 were detected using sheep polyclonal antibodies and HRP conjugated anti-sheep secondary antibody. ECL detection was performed *via* Amersham ECL system (GE Healthcare).

Co-localisation analysis

Fluorescence levels for localisation were measured in MetaMorph using the linescan option set at one pixel width for > 10µm length. Chromatic aberrations were minimized by using a single dichroic filter for all channels.

Checkpoint-silencing assay

Performed exactly as in (Meadows et al., 2011).

Supplemental References

Bahler, J., Wu, J.Q., Longtine, M.S., Shah, N.G., McKenzie, A., 3rd, Steever, A.B., Wach, A., Philippsen, P., and Pringle, J.R. (1998). Heterologous modules for efficient and versatile PCR-based gene targeting in Schizosaccharomyces pombe. Yeast *14*, 943-951.

Keeney, J.B., and Boeke, J.D. (1994). Efficient targeted integration at leu1-32 and ura4-294 in Schizosaccharomyces pombe. Genetics *136*, 849-856.

Krawchuk, M.D., and Wahls, W.P. (1999). High-efficiency gene targeting in Schizosaccharomyces pombe using a modular, PCR-based approach with long tracts of flanking homology. Yeast *15*, 1419-1427.

Meadows, J.C., Shepperd, L.A., Vanoosthuyse, V., Lancaster, T.C., Sochaj, A.M., Buttrick, G.J., Hardwick, K.G., and Millar, J.B. (2011). Spindle checkpoint silencing requires association of PP1 to both Spc7 and kinesin-8 motors. Developmental cell *20*, 739-750.
