# Supplementary material for: Identification of a Sgo2-Dependent but Mad2-Independent Pathway Controlling Anaphase Onset in Fission Yeast
Source: Cell Rep. 2017 Feb 7;18(6):1422–33. doi: 10.1016/j.celrep.2017.01.032 (PMC5316559; doi:10.1016/j.celrep.2017.01.032)
Supplement: Document S2. Article plus Supplemental Information [file mmc3.pdf]

Cell Reports

Article

Identification of a Sgo2-Dependent but Mad2-Independent Pathway Controlling Anaphase Onset in Fission Yeast

Graphical Abstract

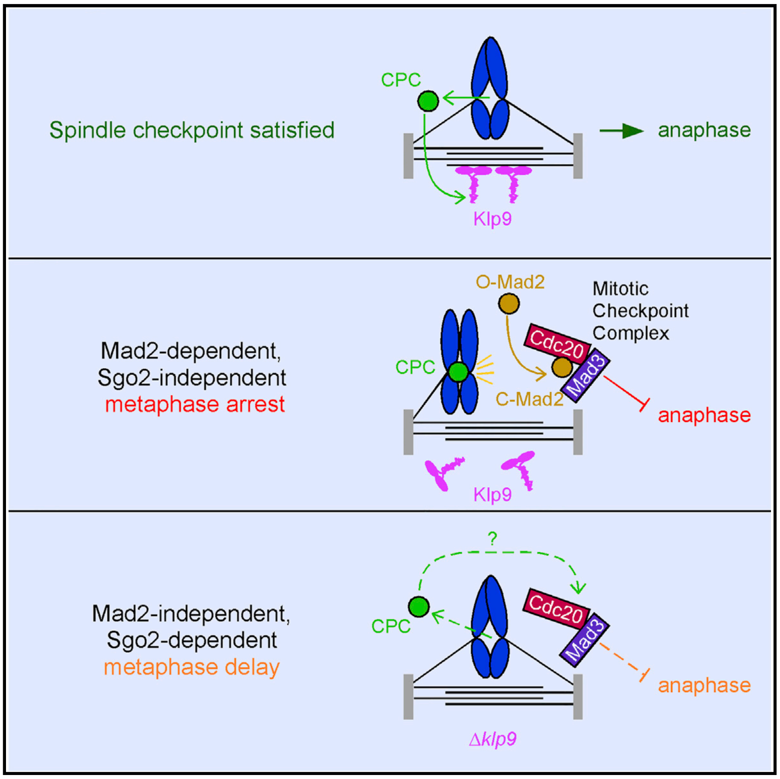

Highlights

- Klp9 (kinesin-6) is required for re-localization of CPC to the spindle midzone
- Klp9 plays a motor-independent role in controlling the timing of anaphase onset
- Interaction of CPC and Klp9 terminates Sgo2-dependent inhibition of the APC/C
- Mad3-Slp1 (Cdc20) is a bona fide inhibitor of APC/C in vivo

Authors

John C. Meadows, Theresa C. Lancaster, Graham J. Buttrick, ..., Maria del Mar Mora-Santos, Kevin G. Hardwick, Jonathan B.A. Millar

Correspondence

j.millar@warwick.ac.uk

In Brief

Meadows et al. find that redistribution of the chromosome passenger complex (CPC) from centromeres to Klp9/MKLP2 at the spindle midzone terminates a Sgo2-dependent pathway controlling APC/C activation. Their data indicate that redistribution of CPC and Klp9 terminates a Mad2-independent, APC/C-inhibitory pathway that is distinct from the spindle assembly checkpoint.

Meadows et al., 2017, Cell Reports *18*, 1422–1433
February 7, 2017 © 2017 The Author(s).
http://dx.doi.org/10.1016/j.celrep.2017.01.032

CellPress

OPEN ACCESS
CellPress

Cell Reports
Article

Identification of a Sgo2-Dependent but Mad2-Independent Pathway Controlling Anaphase Onset in Fission Yeast

John C. Meadows,[1,2] Theresa C. Lancaster,[1] Graham J. Buttrick,[1] Alicja M. Sochaj,[3] Liam J. Messin,[1] Maria del Mar Mora-Santos,[1] Kevin G. Hardwick,[3] and Jonathan B.A. Millar[1,4,*]
[1]Division of Biomedical Sciences, Warwick Medical School, University of Warwick, Coventry CV4 7AL, UK
[2]Institute of Advanced Study, University of Warwick, Coventry CV4 7AL, UK
[3]Wellcome Trust Centre for Cell Biology, University of Edinburgh, Edinburgh EH9 3JR, UK
[4]Lead Contact
*Correspondence: j.millar@warwick.ac.uk
http://dx.doi.org/10.1016/j.celrep.2017.01.032

SUMMARY

The onset of anaphase is triggered by activation of the anaphase-promoting complex/cyclosome (APC/C) following silencing of the spindle assembly checkpoint (SAC). APC/C triggers ubiquitination of Securin and Cyclin B, which leads to loss of sister chromatid cohesion and inactivation of Cyclin B/Cdk1, respectively. This promotes relocalization of Aurora B kinase and other components of the chromosome passenger complex (CPC) from centromeres to the spindle midzone. In fission yeast, this is mediated by Clp1 phosphatase-dependent interaction of CPC with Klp9/MKLP2 (kinesin-6). When this interaction is disrupted, kinetochores bi-orient normally, but APC/C activation is delayed via a mechanism that requires Sgo2 and some (Bub1, Mph1/Mps1, and Mad3), but not all (Mad1 and Mad2), components of the SAC and the first, but not second, lysine, glutamic acid, glutamine (KEN) box in Mad3. These data indicate that interaction of CPC with Klp9 terminates a Sgo2-dependent, but Mad2-independent, APC/C-inhibitory pathway that is distinct from the canonical SAC.

INTRODUCTION

Anaphase onset is initiated when all chromosomes have been correctly bi-oriented. This dependence is imposed by the spindle assembly checkpoint (SAC), which prevents activation of the anaphase-promoting complex/cyclosome (APC/C) when kinetochores are either unattached or not under tension (Lara-Gonzalez et al., 2012). Components of the SAC include Mad1, Mad2, Mad3(BubR1), and Bub3 and the kinases Bub1, Mph1(Mps1), and Aurora B. SAC proteins, with the exception of centromere-bound Aurora B kinase, are recruited to kinetochores that are tensionless or not bound to spindle microtubules. Association of Mad2 with a template of kinetochore-bound Mad1-Mad2 induces a conformational change in Mad2 that promotes its association with Mad3(BubR1), Bub3, and Cdc20 to form the mitotic checkpoint complex (MCC), which is a potent inhibitor of the APC/C (Lara-Gonzalez et al., 2012). During prometaphase and metaphase, MCC is continually assembled at the kinetochore and disassembled, by the actions of APC15 and p31^{comet}, to make the cell sensitive to the status of kinetochore-microtubule attachment (Foster and Morgan, 2012; Mansfeld et al., 2011; Miniowitz-Shemtov et al., 2012; Teichner et al., 2011; Uzunova et al., 2012). Following satisfaction of the SAC, Mad1 and Mad2 rapidly dissociate from the final sister kinetochore pair, and the MCC is disassembled, permitting APC/C activation. Although Mad2 is essential for the formation of the MCC, it exists in sub-stoichiometric amounts in the MCC, indicating that another complex composed of just BubR1, Bub3, and Cdc20 (dubbed BBC) also exists in cells (Han et al., 2013; Kulukian et al., 2009; Nilsson et al., 2008; Westhorpe et al., 2011). It is presently unclear whether this latter complex is also an inhibitor of the APC/C or is simply a product of Mad2 removal from the MCC.

Activation of the APC/C results in the ubiquitination and subsequent destruction of Securin and Cyclin B. This leads to cleavage of the Cohesin complex through activation of Separase and inactivation of Cyclin B-Cdk1 kinase, respectively (Lara-Gonzalez et al., 2012). This triggers loss of sister chromatid cohesion and an alteration of microtubule dynamics that enable sister chromatids to move to spindle poles (anaphase A) and spindle poles to move apart (anaphase B). Despite our understanding of the underlying biochemistry, it remains unclear how these events are co-ordinated in time and space. In addition to altering microtubule dynamics, inactivation of Cdk1/Cyclin B prevents re-activation of the SAC during anaphase (Rattani et al., 2014; Vázquez-Novelle et al., 2014). This is mediated in part by relocalization of Aurora B kinase and other components of the chromosome passenger complex (CPC), including Survivin, inner centromere protein (INCENP), and Borealin, from centromeres to the spindle midzone (Carmena et al., 2012; Mirchenko and Uhlmann, 2010; Vázquez-Novelle and Petronczki, 2010). In human cells, relocalization of the CPC requires dephosphorylation of INCENP on Cdk1 phosphorylation sites and interaction of CPC with MKLP2 (mitotic kinesin-like protein 2), a kinesin-6 that binds microtubules at the central spindle (Gruneberg et al.,

 © 2017 The Author(s).
This is an open access article under the CC BY license (http://creativecommons.org/licenses/by/4.0/).

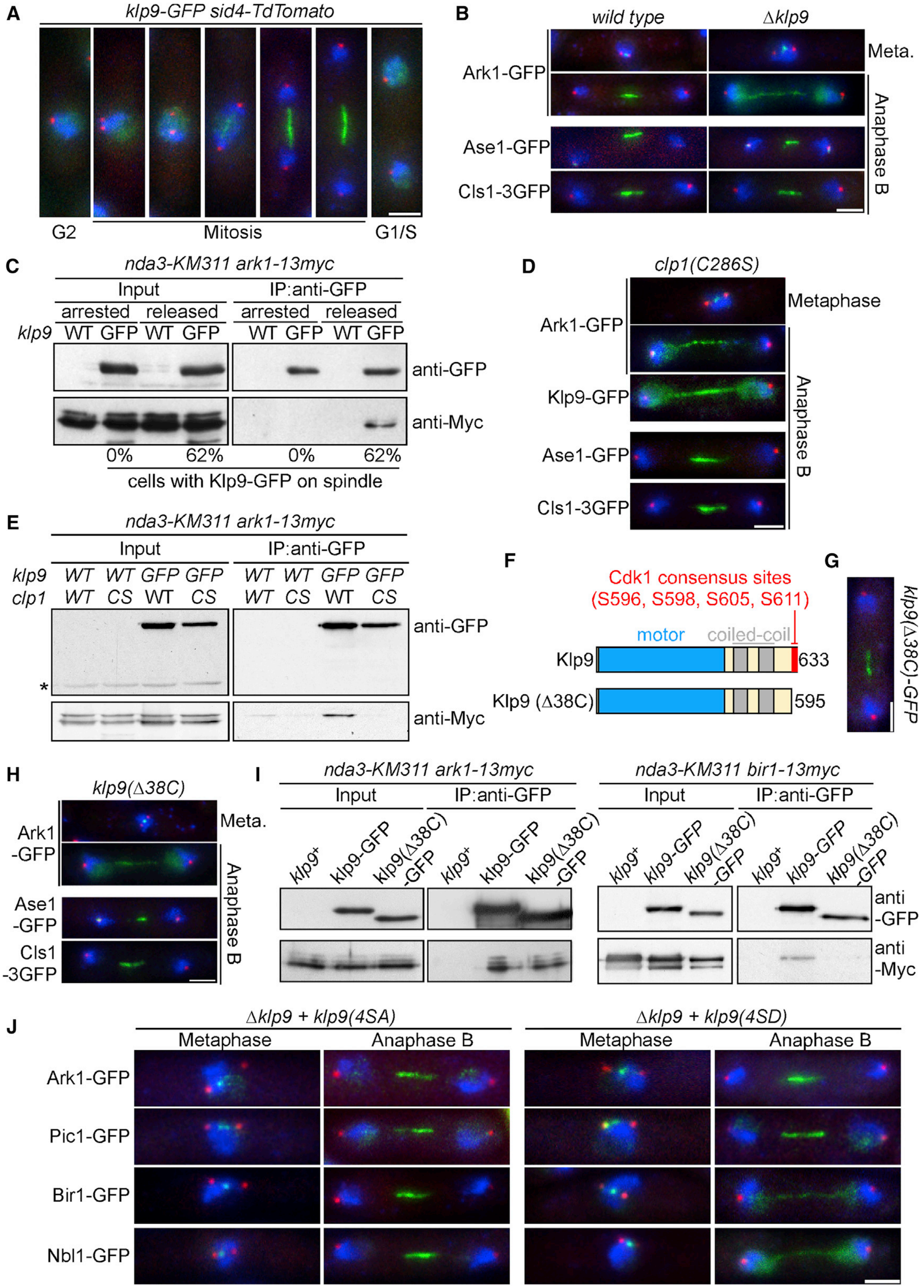

Figure 1. CPC Relocalization to the Spindle Midzone Requires Interaction with Dephosphorylated Klp9

(A) Cells expressing Klp9-GFP (green) and Sid4-TdTomato (spindle pole bodies, red throughout). Chromatin is stained with DAPI (blue throughout).

(B) Ark1 relocalization requires Klp9. Ark1-GFP, Ase1-GFP, and Cls1-3GFP localization (green) was examined in the presence (wild type) and absence (*Δklp9*) of Klp9.

(legend continued on next page)

2004; Hümmer and Mayer, 2009; Kitagawa et al., 2014; Lee et al., 2010). In this study, we examine the factors required for relocalization of the CPC in fission yeast. In doing so, we have uncovered an unexpected non-catalytic role for the Klp9 (kinesin-6) motor protein in terminating a Sgo2-dependent, but Mad2-independent, pathway controlling the timing of APC/C activation that is distinct from the canonical SAC.

RESULTS

Dephosphorylation of Klp9 Promotes Relocalization of CPC to the Spindle Midzone

Fission yeast contains a single member of the kinesin-6 family, Klp9, which localizes to the nucleoplasm during interphase, prometaphase, and metaphase and to the spindle midzone during anaphase B, where it co-localizes with Ark1 (Aurora B), Ase1 (MAP65/PRC1), and Cls1/Peg1 (CLASP) (Figure 1A; Figure S1A; data not shown). We find that Ark1 and all other components of the CPC, including Nbl1 (Borealin), Pic1 (INCENP), and Bir1 (Survivin), fail to relocalize to the spindle midzone during anaphase B in the absence of Klp9, although centromere association of CPC components during metaphase is unaffected (Figure 1B; Figures S1B and S1C). This effect is specific because spindle midzone localization of Ase1 and Cls1/Peg1 during anaphase B does not require Klp9. Consistent with the notion that Klp9 is the structural and functional equivalent of mammalian MKLP2, we find that Klp9 interacts with Ark1 during anaphase B but not during metaphase (Figure 1C).

Localization of both Klp9 and Ark1 to the spindle midzone is promoted by chemical inactivation of Cdc2/Cdk1 kinase (Dischinger et al., 2008) in cells arrested in metaphase by overexpression of the Mph1 spindle checkpoint kinase (Figures S1D and S1E). This suggests that, like MKLP2, relocalization of Klp9 and CPC at the spindle midzone is triggered by reversal of Cdk1-dependent phosphorylation (Hümmer and Mayer, 2009). Consistently, we find that both Klp9 and the CPC fail to concentrate at the anaphase spindle midzone in *clp1(C286S)* cells, which express a catalytically inactive allele of the Clp1 (CDC14) phosphatase (Wolfe et al., 2006). Ase1 and Cls1/Peg1 localization to the spindle midzone is not disrupted in *clp1(C286S)* cells, suggesting that the spindle midzone remains intact (Figure 1D). Consistent with this, interaction between Klp9 and Ark1 in anaphase is abolished in the absence of Clp1 phosphatase activity (Figure 1E). Phospho-proteomic studies have revealed that Klp9 contains four phosphorylation sites (S596, S598, S605, and S611) for Cdk1/Cdc2 kinase in its C-terminal 38 amino acids (Chen et al., 2013). To examine whether these residues are involved in either Klp9 and/or CPC relocalization and Klp9-CPC interaction, we constructed a Klp9(Δ38C) mutant that lacked the 38 C-terminal residues (Figure 1F). The Klp9(Δ38C) protein, although reduced in interphase nuclear accumulation, was expressed at wild-type levels and recruited normally to the anaphase B spindle midzone, indicating that dephosphorylation of the C-terminal Cdk1 sites in Klp9 is not necessary for its association to the anaphase B spindle midzone (Figures 1G and 1I; Figure S1F). Importantly, however, concentration of the CPC at the spindle midzone is abolished in *klp9(Δ38C)* cells. This is not due to general disruption of the spindle midzone because Ase1 and Cls1/Peg1 localization during anaphase B were unaffected in *klp9(Δ38C)* cells (Figure 1H; Figures S1B and S1C). Notably, although the C-terminal 38 amino acids of Klp9 were required for its interaction with Bir1 during anaphase B, the interaction between Klp9 and Ark1 remained unaffected (Figure 1I). This indicates that the C terminus of Klp9 is required to interact with some CPC components but not others. To examine this further, we mutated the four C-terminal serine residues (S596, S598, S605, and S611) in Klp9 to either alanine to create a non-phosphorylatable mutant (*klp9(4SA)*) or to aspartic acid to mimic the phosphorylated state (*klp9(4SD)*). Although Klp9 phospho-mutant proteins display similar expression levels and localize normally to the spindle midzone (Figure S1G), we find that Bir1 and Nbl1 bind the spindle midzone in *klp9(4SA)* cells but not *klp9(4SD)* cells, demonstrating that dephosphorylation of these residues is required for spindle midzone accumulation of these CPC components (Figure 1J). By contrast, localization of the CPC components Ark1 and Pic1 is unaltered in either *klp9(4SA)* and *klp9(4SD)* cells. We therefore conclude that CPC relocalization in anaphase B depends on phospho-regulated interaction with Klp9, but different CPC components are recruited by distinct regions of Klp9.

It has been reported previously that dephosphorylation of Klp9 on S596, S598, S605, and S611 triggers its interaction with Ase1 when Cdk1-dependent phosphorylation of Ase1 on S640, S683, S688, and S693 is reversed and, second, that this interaction is important for determining the rate of anaphase B spindle elongation (Fu et al., 2009). Our results prompted us to re-examine

(C) Klp9 and Ark1 physically interact following anaphase onset. *nda3-KM311* cells containing either Klp9-GFP (GFP) or Klp9 (WT) were arrested for 6 hr to synchronize in a prometaphase-like state, and extracts were prepared at the block point (arrested) and 20 min following a shift to the permissive temperature (released). Klp9-GFP on spindles was used to monitor synchrony, and association between Ark1-13Myc and Klp9-GFP was assessed by immunoprecipitation and western blot.

(D) Clp1 activity is required for localization of both Klp9-GFP and Ark1-GFP to the spindle midzone but not Ase1-GFP or Cls1-3GFP (all green).

(E) Clp1 activity is required for the interaction of Klp9 and Ark1. Immunoprecipitation and western blotting conditions were as in (C) for released cells.

(F) Schematic of full-length *S. pombe* Klp9 and a truncated protein, omitting the final four Cdk1 consensus sites.

(G) C-terminally truncated Klp9 localizes to the spindle midzone in anaphase B. Cells expressing Klp9(Δ38C)-GFP (green) were grown to mid-log phase and fixed.

(H) Ark1 relocalization requires the Klp9 C terminus. Ark1-GFP, Ase1-GFP, and Cls1-3GFP localization (green) was examined in the absence of the last 38 amino acids of Klp9.

(I) The Klp9 C terminus is required for physical interaction with the CPC component Bir1 but not Ark1. Immunoprecipitation and western blotting conditions were as in (C) for released cells.

(J) Cells containing only phospho-mimetic Klp9 (*Δklp9 + klp9(4SD)*) correctly relocalize the CPC components Ark1-GFP and Pic1-GFP but not Bir1-GFP and Nbl1-GFP (green) to the spindle midzone, whereas relocalization is unaffected in the non-phosphorylatable version (*Δklp9 + klp9(4SA)*).

Scale bars, 2 μm. See also Figures S1 and S2.

these conclusions. We find that the rate of spindle pole separation in anaphase B in *klp9(4SD) ase1(4SD)* cells (0.95 ± 0.07 μm/min) is only marginally slower than in the equivalent wild-type cells (1.2 ± 0.14 μm/min) and faster than in *Δklp9* cells (0.68 ± 0.14 μm/min) (Figure S2A). Moreover, we find no difference in the rate of spindle pole separation in *Δclp1* or *clp1(C286S)* cells (in cells not displaying lagging sister chromatids) compared with that observed in wild-type cells, although the frequency of anaphase B spindle collapse increases significantly (Figure S2B). These differences may be because previous studies used GFP-tagged tubulin, which is known to alter normal microtubule dynamics and influence the efficiency of chromosome segregation, whereas we have monitored spindle elongation using fluorescently tagged kinetochores and spindle poles. Perhaps more significantly, we failed to detect an interaction between Ase1 with Klp9 during anaphase B (data not shown), although we observed a strong interaction between Ase1 and Cls1 by co-immunoprecipitation and two-hybrid analysis, as observed previously (Bratman and Chang, 2007). Taken together, our data indicate that de-phosphorylation of the C terminus of Klp9 by Clp1 phosphatase promotes interaction of CPC components with Klp9 at the spindle midzone rather than interaction of Klp9 with Ase1 to influence the rate of spindle elongation.

Klp9 Is Required for Timely Anaphase Onset after Chromosome Bi-orientation

To examine the effect of Klp9 on mitotic progression, we monitored kinetochore and spindle pole dynamics in living cells. Consistent with the observations of Fu et al. (2009), we find that the average rate of spindle pole separation during anaphase B is approximately half that in cells lacking Klp9 (Figure 2A). However, we also found that the average duration of prometaphase and metaphase in Δ*klp9* cells is greater than in wild-type cells (Figure 2A; Figure S3A). Additionally, we noted that metaphase spindles in Δ*klp9* cells sometimes collapse prior to the onset of anaphase, particularly when anaphase onset is substantially delayed. Importantly, Cdc13 (Cyclin B) remains bound to spindles and separated spindle poles for longer in *Δklp9* cells than in wild-type cells, indicating that Klp9 is required for timely APC/C activation (Figure S3B). It is well known that APC/C activation is inhibited when microtubule-kinetochore interaction is perturbed, such as in cells lacking Dam1 (Dam1, Ask1, Spc34, Hsk3 [DASH] complex) or Klp5/Klp6 (kinesin-8) (Sanchez-Perez et al., 2005). However, Δ*klp9* cells display no defect in chromosome segregation, as judged by a mini-chromosome loss assay, nor do they display altered sensitivity or resistance to thiabendazole (TBZ), a microtubule-depolymerizing agent (Figures 2B and 2C). Moreover, Klp9 is not required for recovery of cells from a metaphase arrest imposed by an *nda3-KM311* (β-tubulin) mutation, nor is Klp9 required for silencing the spindle checkpoint when the checkpoint is over-ridden by chemical inhibition of Ark1 kinase in *nda3-KM311 ark1-as3* cells (Figures S3C and S3D). Instead, we find that sister kinetochores become properly bi-oriented during prometaphase and metaphase in *Δklp9* cells, as judged by continued separation of Cen2-GFP signals along the spindle axis, but remain in this configuration for significantly longer than in wild-type cells even though the spindle length at anaphase onset in *Δklp9* cells is similar to that in wild-type cells at anaphase onset (Figure 2D; Figure S3E). Taken together, these results suggest that Klp9 functions after, or separately from, chromosome bi-orientation to dictate the timing of APC/C activation.

The Non-catalytic C Terminus of Klp9 Controls the Timing of Anaphase Onset

Because Klp9 is a molecular motor that drives anti-parallel microtubules apart, we hypothesized that its association with the spindle midzone may trigger APC/C activation by increasing inter-kinetochore tension just prior to the onset of anaphase. To address this possibility, we generated a rigor mutant by changing glycine 296 to alanine in the switch II region of the kinesin motor domain (Browning et al., 2003; Figure 3A). This mutant, termed Klp9(SwII), was expressed at wild-type levels and localizes to the entire anaphase B spindle rather than just the spindle midzone (Figures S4A and S4B). Moreover, CPC components localize to the entire anaphase B spindle when Klp9 motor activity is absent (Figure S4C). Importantly, re-introduction of full-length *klp9* or truncated *klp9(Δ38C)*, but not the catalytically inactive *klp9(SwII)* mutant, restores the wild-type rate of anaphase B spindle elongation in *Δklp9* cells (Figure 3B), indicating that motor activity, but not the C terminus of Klp9, is required for anaphase spindle elongation. In direct contrast, re-introduction of wild-type *klp9* or *klp9(SwII)*, but not *klp9(Δ38C)*, rescues the delay in anaphase onset in *Δklp9* cells, indicating that the C-terminal 38 amino acids, but not the motor activity of Klp9, dictates the timing of anaphase onset (Figures 3B and 3C). Consistently, the percentage of cells in prometaphase and metaphase in *klp9(4SD)* cells (6.0% ± 0.7%) is similar to that observed in *Δklp9* cells (6.9% ± 0.8%) and greater than that observed in wild-type cells (3.8% ± 0.8%) (Figure S3A). To determine whether this is due to delayed APC/C activation, we monitored Cdc13 (Cyclin B) levels in live cells. We find that re-introduction of wild-type *klp9* or *klp9(SwII)*, but not *klp9(Δ38C)*, restores the timing of Cdc13 destruction in *Δklp9* cells (Figure 3D; Figure S4D). These results indicate that Klp9 has at least two separable roles during mitosis: a motor-dependent role in determining the rate of spindle elongation and a motor-independent role in determining the timing of APC/C activation; the latter relies on interaction of the chromosome passenger complex with the non-catalytic C terminus of Klp9.

Loss of Klp9 Delays Anaphase Onset via a Sgo2-Dependent but Mad2-Independent Pathway

We next examined whether the delay in APC/C activation in *Δklp9* cells is dependent on components of the SAC by monitoring the percentage of cells in prometaphase and metaphase in log-phase cell populations. Surprisingly, we find that the delay in anaphase onset in *Δklp9* cells is dependent on Bub1, Mph1, and Mad3 but entirely independent of Mad1, Mad2, or Bub3 (Figure 4A). To confirm this, we monitored the duration of prometaphase and metaphase in live cells and found that the anaphase delay observed in *Δklp9* cells is abolished when Mad3, but not Mad2, is deleted (Figure 4B). Consistently, although Mad2 dimerization is a prerequisite for spindle checkpoint function (De Antoni et al., 2005; Mapelli et al., 2006; Zich et al., 2012), the delay over anaphase onset in *Δklp9* cells occurs even in

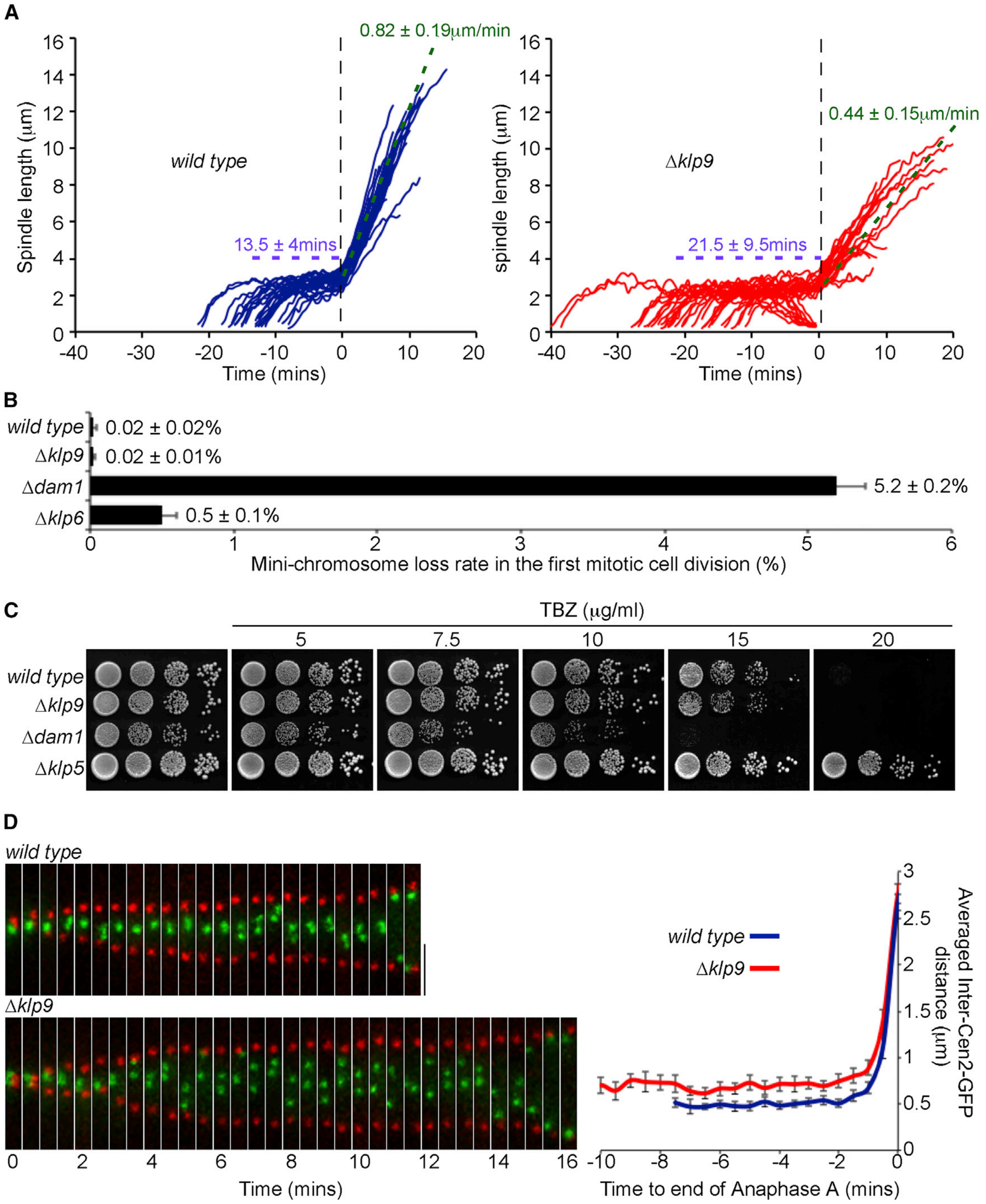

Figure 2. Klp9 Is Required for Timely Anaphase Onset and Regulation of the Anaphase B Spindle Elongation Rate and Inter-centromere Distance but Not for Fidelity of Chromosome Segregation

(A) Log-phase *ndc80-GFP cdc11-CFP* (left, n = 29) or *Δklp9 ndc80-GFP cdc11-CFP* (right, n = 32) cells were imaged by fluorescence microscopy. Spindle length in individual mitotic cells was calculated at 30-s intervals. The completion of anaphase A was taken as T = 0 for each movie. Spindle collapses are traces in which spindle length reduces to zero. Average times in prometaphase and metaphase and anaphase B spindle elongation rate are shown ± SD.

(B) Klp9 is not required for proficient maintenance of a minichromosome. Error bars show standard deviation.

(C) 10-Fold serial dilutions reveal that cells lacking Klp9 (*Δklp9*) are no more sensitive to TBZ than wild-type cells.

(D) Metaphase cells lacking Klp9 have increased distance between sister centromeres for protracted periods. Shown are time-lapse images of cells expressing *cen2-GFP* (green) and *sid4-TdTomato* (red) in the presence (top) or absence (bottom) of Klp9. Scale bar, 2 μm. The graph shows collated data from multiple wild-type (n = 14) and *Δklp9* cells (n = 20). The completion of anaphase A was taken as T = 0 for each movie. Error bars show SEM.

See also Figure S3.

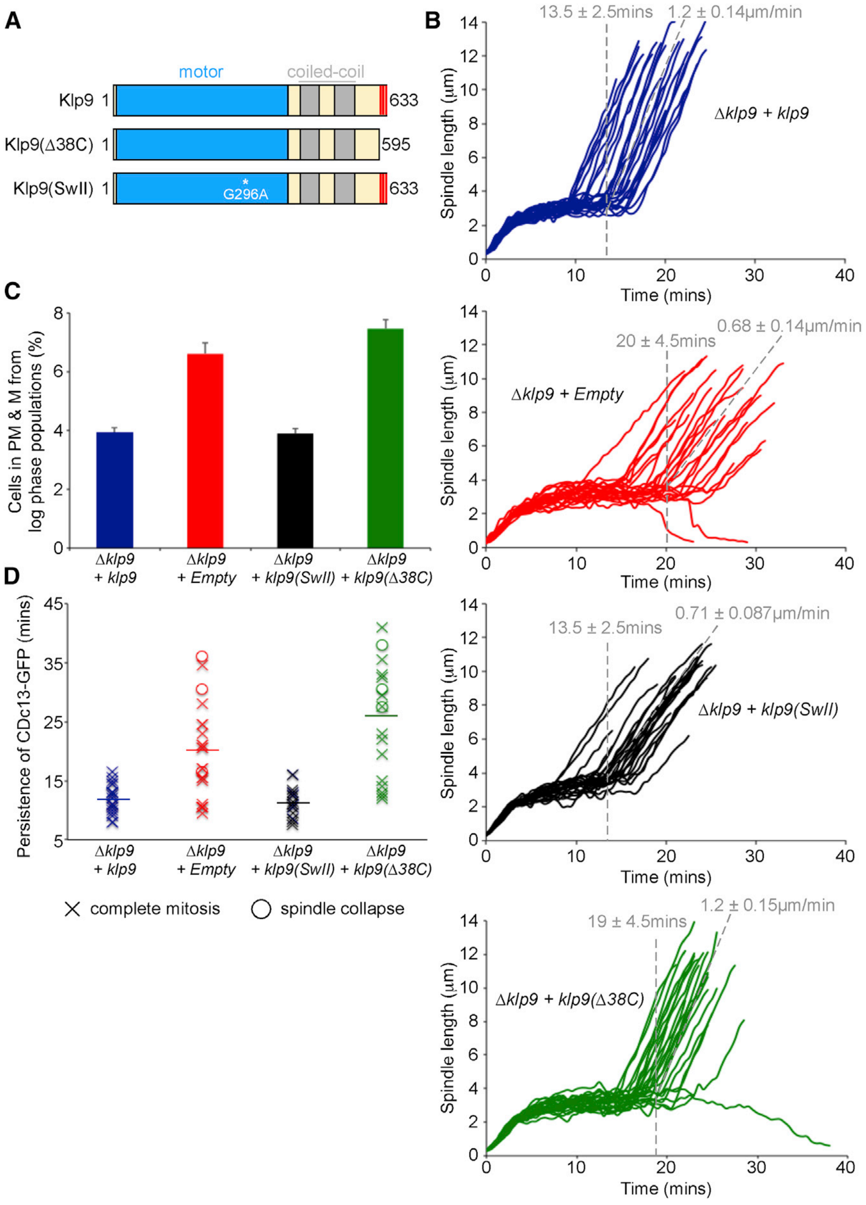

Figure 3. The C Terminus and Motor Activity of Klp9 Have Distinct Roles in Regulating the Timing of Anaphase Onset and Anaphase B Spindle Elongation, Respectively

(A) Schematic of Klp9 proteins used showing full-length, C-terminal truncation, and kinesin domain mutant.

(B) Log phase *dad1-GFP sid4-TdTomato* cells deleted for *klp9* with either *klp9* (first panel), empty plasmid (second panel), *klp9(SwII)* mutant (third panel), or *klp9(Δ38C)* (fourth panel) integrated were imaged by fluorescence microscopy. Spindle length in individual mitotic cells was calculated at 30-s intervals. Movies are aligned at spindle pole body separation. Spindle collapses are traces in which spindle length reduces to zero. n = 25 for each strain. Quantifications of the duration of prometaphase and metaphase and anaphase B spindle elongation rate are shown ± SD.

(C) The cells used in (B) were grown to mid-log phase at 30°C and fixed; the proportion of cells in prometaphase and metaphase (PM & M) was then established by fluorescence microscopy. Error bars indicate SD.

(D) Log-phase *cdc13-GFP* cells deleted for *klp9* with either *klp9*, empty plasmid, *klp9(SwII)* mutant, or *klp9(Δ38C)* integrated were imaged by fluorescence microscopy. The persistence of Cdc13-GFP (Cyclin B) between spindle poles and on spindles was assayed at 30-s intervals in individual mitotic cells. Horizontal lines show mean values.

See also Figure S4.

mad2 dimerization mutants (Figure 4C). By contrast, the anaphase delay in cells lacking Dam1 and Mal3 (EB1) is dependent on Mad1, Mad2, Mad3, and Bub1, consistent with the notion that the canonical kinetochore attachment checkpoint is activated in these cells (Figures 4C and 4D).

We next sought factors that are required for the anaphase delay in *Δklp9* cells but dispensable for the canonical response to unattached kinetochores. The Bub1 protein contains several domains that are required for spindle checkpoint signaling and chromosome bi-orientation. These include a conserved middle region (Bub1-cm1) that, when phosphorylated by Mph1 kinase, recruits Mad1 and Mad2 to delay anaphase onset in response to unattached kinetochores (Heinrich et al., 2014; Mora-Santos et al., 2016). Consistently, we find that *Δmal3 bub1-SATATA* mutant cells, which express a mutant that fails to recruit Mad1 and Mad2 to Bub1, display no delay over anaphase onset (Figure 4D). By contrast, the delay in anaphase onset in *Δklp9 bub1-SATATA* cells is unaffected, consistent with our finding that neither Mad1 nor Mad2 are required for delaying anaphase onset in the absence of Klp9 (Figure 4D). Bub1 additionally contains a kinase domain that phosphorylates histone H2A to recruit Sgo2 (Shugoshin protein 2), which, together with a separate pathway involving Haspin kinase, recruits the CPC to centromeric heterochromatin (Yamagishi et al., 2010). It has been shown previously that Sgo2 is required for the delay in anaphase onset in temperature-sensitive cohesin mutants, presumably by enabling CPC to correct syntelic attachments, but not required for metaphase arrest in the absence of spindle microtubules (Kawashima et al., 2007). Consistently, we find that the delay in anaphase onset in *Δdam1* and *Δmal3* cells is not dependent on Sgo2. In stark contrast, however, the delay in anaphase onset in *Δklp9* is strictly dependent on the presence of Sgo2 (Figure 4D). Live analysis of *ark1-GFP* cells shows that Aurora B levels at inner centromeres are significantly reduced, but not abolished, in cells lacking Sgo2, as observed previously (Kawashima et al., 2007; Vanoosthuyse et al., 2007), but relocalization of Ark1 to the spindle midzone in anaphase B was unperturbed (Figure 4E).

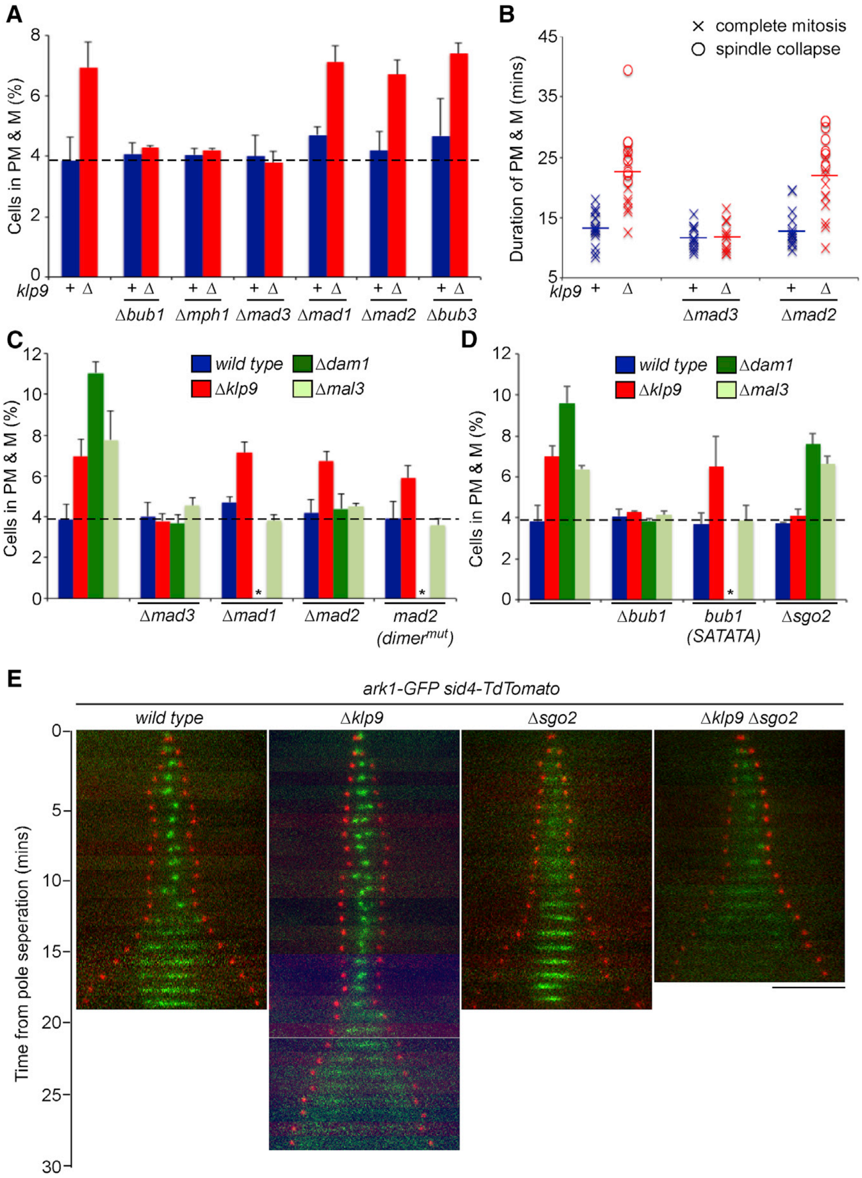

Figure 4. A Mad2-Independent, but Sgo2-Dependent, Pathway Delays Anaphase Onset in the Absence of Klp9

(A) The delay over anaphase onset in the absence of Klp9 is abrogated by deletion of some, but not all, SAC proteins. *dad1-GFP sid4-TdTomato* cells were grown to mid-log phase and fixed; the proportion of cells in PM & M was then determined by fluorescence microscopy. Cells with either wild-type Klp9 (+) or a total deletion (Δ) were tested against a panel of SAC component deletions. Error bars correspond to SD throughout.

(B) The Klp9-mediated delay over anaphase onset is dependent on Mad3 but independent of Mad2. The indicated cells from (A) were grown to mid-log phase, and the duration of PM & M was assayed by live-cell analysis. Horizontal lines show mean values.

(C) Mad1 and Mad2 are not required to delay anaphase onset specifically in the absence of Klp9. *dad1-GFP sid4-TdTomato* cells, either wild-type or deleted for Klp9, Dam1, or Mal3, were tested for their requirements of Mad1, Mad2, and Mad2 dimerization and Mad3 in maintaining a mitotic delay. Asterisks indicate that *Δdam1 Δmad1* and *Δdam1 mad2(dimer^mut)* cells were too sick to accurately assess.

(D) Sgo2 is required to delay anaphase onset in the absence of Klp9. Mitotic profiles of *dad1-GFP sid4-TdTomato* cells, either wild-type or deleted for Klp9, Dam1, or Mal3, were established from log-phase cultures in the presence and absence of Bub1, Bub1(SATATA), and Sgo2. Asterisks indicate that *Δdam1 Δbub1(SATATA)* was synthetically lethal in this background.

(E) Effect of deleting Klp9 and Sgo2 on CPC localization and the timing of anaphase onset. *ark1-GFP sid4-TdTomato* cells in the presence or absence of Klp9 or Sgo2 or both were grown to mid-log phase at 30°C and then imaged live by fluorescence microscopy. Scale bar, 5 μm.

In cells lacking both Sgo2 and Klp9, Ark1 bound centromeres weakly before anaphase onset but failed to bind the spindle midzone after anaphase onset, confirming that Klp9 and Sgo2 influence distinct aspects of CPC localization during M phase (Figures 4D and 4E).

Mad3-Slp1 (Cdc20) Inhibits APC/C in the Absence of Klp9

We next explored how the APC/C is inhibited in the absence of Klp9. The Mad3(BubR1) family proteins contain two lysine, glutamic acid, glutamine (KEN) boxes that are both required for inhibition of APC/C in response to unattached kinetochores. The N-terminal KEN-20 box interacts directly with MCC^{Cdc20} and is required for MCC assembly, whereas the second KEN-271 box is dispensable for MCC assembly (Chao et al., 2012; Sczaniecka et al., 2008) but, instead, is required for interaction of MCC^{Cdc20} with a second Cdc20 molecule bound to APC/C (Izawa and Pines, 2015; Lara-Gonzalez et al., 2011). We confirmed that both KEN boxes are required for delaying anaphase onset in response to unattached kinetochores in *nda3-KM311*, *Δdam1*, and *Δmal3* cells (Figure 5A; Figures S5A–S5C). Strikingly, however, we find that the delay in anaphase onset in *Δklp9* cells is dependent on Mad3-KEN20 but entirely independent of Mad3-KEN271 (Figure 5A). Taken together, we reasoned that the delay over APC/C activation in *Δklp9* cells must be dependent on the generation of an APC/C inhibitor that contains just Mad3 and Slp1 (Cdc20) but not Mad2. To test this, Mad3 was immunoprecipitated from lysates of *Δklp9 cdc25-22* and *Δmad2 Δklp9 cdc25-22* cells, and the association of Slp1 (Cdc20) with Mad3 was tested by western blot. Strikingly, we find that Mad3 interacts with Slp1 in a cell cycle- and KEN20-dependent manner, both in the presence or absence of Mad2, although, in the latter case, the interaction is somewhat weaker (Figure 5B). Taken together, these findings confirm the

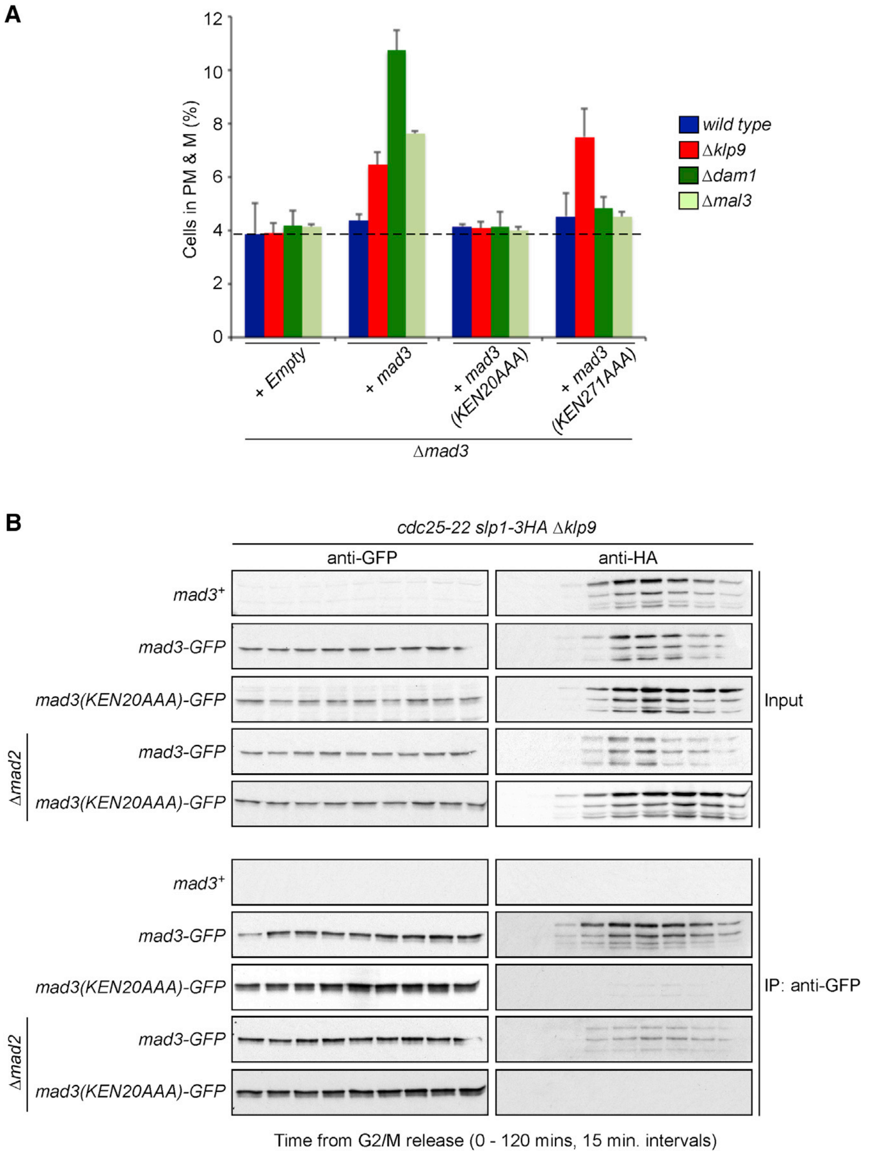

Figure 5. The First, but Not Second, Mad3 KEN Box Is Required to Delay Anaphase Onset and to Interact with Slp1 in the Absence of Klp9 and Mad2

(A) In the absence of Klp9, the first, but not second, Mad3 KEN box is required to delay anaphase onset. The histogram shows the effect of deleting Klp9, Dam1, or Mal3 on the mitotic profiles of *dad1-GFP sid4-TdTomato* cells deleted for Mad3 expressing either empty vector, wild-type Mad3, or Mad3 with either the first or second KEN box mutated. Error bars show SD.

(B) In the absence of Klp9, Mad3 can interact with Slp1 without Mad2 via KEN20. *cdc25-22 slp1-3HA Δklp9* cells containing either untagged Mad3 or Mad3-GFP or Mad3(KEN20AAA)-GFP in the presence and absence of Mad2 were arrested for 4 hr at 35.5°C to synchronize at the G2/M transition. Following release at 25°C, extracts were prepared every 15 min. Association between Slp1-3HA and Klp9-GFP was assessed by immunoprecipitation and western blot.

See also Figure S5.

importance of the second KEN box in Mad3/BubR1 in the inhibition of APC/C by MCC but, more importantly, provide compelling evidence that a Mad3-Slp1 (Cdc20) complex that lacks Mad2 is also an authentic inhibitor of APC/C in vivo.

DISCUSSION

In this study, we demonstrate that fission yeast Klp9 displays strikingly similar characteristics to human MKLP2, in that both interact with the CPC and are required for CPC concentration at the spindle midzone during anaphase. In human cells, phospho-dependent interaction of CPC with MKLP2 is thought to prevent SAC re-activation after anaphase (Vázquez-Novelle and Petronczki, 2010). However, our data suggest that, perhaps in addition to this function, phospho-dependent interaction of CPC with Klp9 terminates a Sgo2-dependent, but Mad2-independent, pathway controlling the timing of anaphase onset. We provide a model to explain how this might occur (Figure 6). During prometaphase and metaphase, interaction of CPC with centromeric chromatin is maintained by Cdk1-dependent phosphorylation of Survivin, which triggers its interaction with Sgo2 (Tsukahara et al., 2010). In fission yeast, the Clp1 phosphatase is released from the nucleolus at the G2/M transition, but its activity is suppressed by Cdk1 phosphorylation during prometaphase and metaphase (Esteban et al., 2004; Wolfe and Gould, 2004; Wolfe et al., 2006). Notably, fission yeast Cig1 cyclin, like human Cyclin A, is destroyed somewhat earlier than Cdc13 (Cyclin B) in early mitosis (Blanco et al., 2000; Kabeche and Compton, 2013). Destruction of Cig1 during late prometaphase may trigger initial dephosphorylation of both Survivin and other components of the CPC, release of the CPC from centromeres, and dephosphorylation of Klp9 to trigger interaction of the CPC with Klp9 and relocalization of the proteins to the spindle midzone. Engagement of Klp9 with the spindle midzone promotes the onset of anaphase B, whereas Klp9 interaction with the CPC terminates Mad3-dependent inhibition of the APC/C, thus promoting destruction of Cdc13 (Cyclin B) and further activation of Clp1 and so on. We suggest that, in this manner, Klp9 co-ordinates the onset of anaphase A and anaphase B in both space and time. When the canonical spindle checkpoint pathway is activated (such as in *nda3-KM311* cells at restrictive temperature), however, Mad3-Slp1 (Cdc20) may be recruited into the MCC (Mad2-Mad3-Slp1). In this situation, silencing of the canonical SAC is, instead, dependent on association of type 1

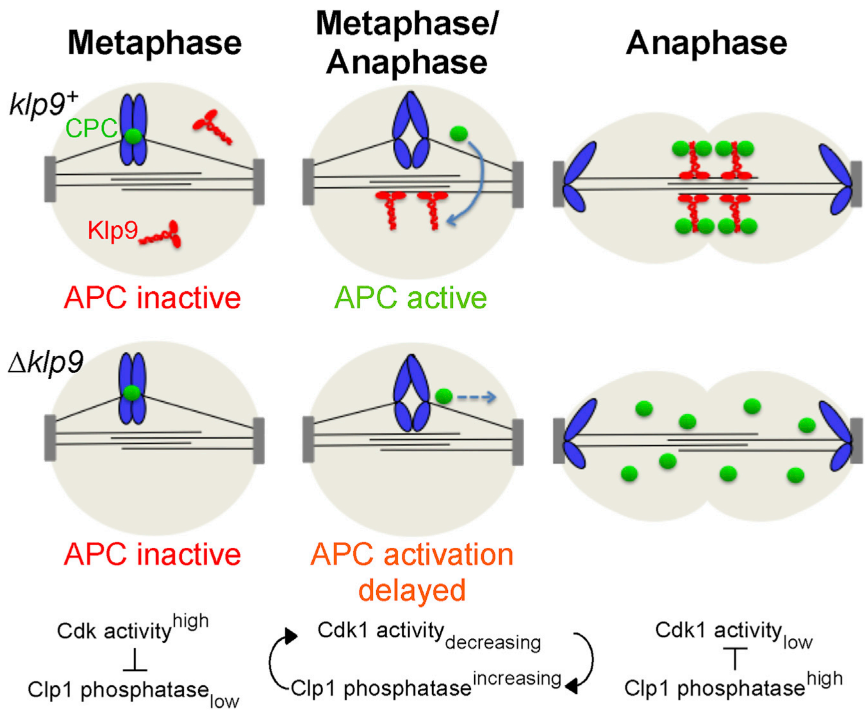

Figure 6. Model to Explain the Role of Klp9 in Sharpening the Anaphase Switch
This is described in the Discussion.

phosphatase (PP1) with both the Spc7/KNL1 kinetochore protein and kinesin-8 motors (Meadows et al., 2011) but not interaction of the CPC with Klp9.

Notably, a previous study reported that overexpression of a non-phosphorylatable Klp9 causes chromosome mis-segregation and lagging sister chromatids during anaphase B (Choi and McCollum, 2012). The authors concluded that Klp9-dependent spindle elongation forces may help correct merotelic kinetochore attachments. Our finding that Klp9 interacts with the CPC necessitates a re-assessment of these conclusions. Intriguingly, re-introduction of either *klp9(SwII)* or *klp9(Δ38C)* mutants rescued the synthetic lethality of *Δase1 Δklp9* mutants (Fu et al., 2009). This suggests that Klp9 plays three separate roles during mitosis: motor-dependent, but tail-independent, control of the anaphase spindle elongation rate; motor-independent, but tail-dependent, control of the timing of anaphase onset; and motor-independent and tail-independent stabilization of the spindle midzone. We also noted clear differences in the behavior of the Ark1-Pic1 (Aurora B-INCENP) and Nbl1-Bir1 (Borealin-Survivin) sub-complexes of the CPC. Although interaction of Ark1 with Klp9 was abrogated in *clp1(C286S)* cells, Ark1 and Pic1 correctly relocalized to the anaphase spindle in *klp9(S4D)* cells whereas Bir1 and Nbl1 did not. This suggests that dephosphorylation of another CPC component(s), for example Pic1, contributes to interaction of the CPC with Klp9. Further experiments will be needed to decipher these mechanisms.

The main inhibitor of the APC/C, the MCC, has been studied extensively in multiple organisms. However, Mad2 is sub-stoichiometric in this complex, indicating that a distinct complex that lacks Mad2, BBC, also exists in human cells (Han et al., 2013; Kulukian et al., 2009; Nilsson et al., 2008; Westhorpe et al., 2011). Although BubR1 is a weak inhibitor of the APC/C in vitro (Kaisari et al., 2016; Tang et al., 2001), there is no evidence that BBC is an inhibitor of the APC/C in vivo. Indeed, BBC may simply result from the removal of Mad2 from the MCC by the action of the p31^{comet}-TRIP13 alanine, alanine, alanine (AAA)-ATPase (Westhorpe et al., 2011). Furthermore, some studies have suggested that Mad2 is required for interaction of BubR1 and Cdc20 and, thus, formation of the MCC (Kulukian et al., 2009; Nilsson et al., 2008). We provide compelling genetic and biochemical evidence that, first, the Mad3-Slp1 (Cdc20) complex can be formed in the absence of Mad2 and, second, that this complex is a bona fide inhibitor of the APC/C in fission yeast. Because the Mad3-Cdc20 complex is generated in an unperturbed cell cycle (Figure S5D), its presence alone cannot be sufficient to delay anaphase onset via inhibition of the APC/C. Notably, we demonstrate that generation of this alternative, Mad2-independent APC/C inhibitory signal requires Sgo2 and the Bub1 and Mph1 kinases. One possibility is that centromere-associated Sgo2-CPC (Aurora B) kinase phosphorylates the Mad3-Slp1 complex to promote its function as an inhibitor of the APC/C. This is not unreasonable because Ipl1 (Aurora B) kinase phosphorylates Mad3 to delay anaphase onset in response to a lack of spindle tension in budding yeast (King et al., 2007). The Mph1 kinase may simply be required to phosphorylate the methione, glutamic acid, leucine, threonine (MELT) motifs of Spc7/KNL1 to target Bub1 kinase to kinetochores, which itself may only be required to phosphorylate histone H2A to load Sgo2-CPC to centromeres (Kawashima et al., 2010). Alternatively, or additionally, the Mph1 and Bub1 kinases may be required to phosphorylate the Mad3-Slp1 complex to promote its function as an inhibitor of the APC/C. Notably, the MCC is also periodically formed in unperturbed mitosis (Zich et al., 2016). We have shown previously that phosphorylation of both Mad2 and Mad3 components by Mph1 kinase is needed for inhibition of the APC/C by the MCC (Zich et al., 2012, 2016). Further experiments will be needed to test whether these same modifications are needed for action of the Mad3-Slp1 (Cdc20) inhibitor. Because the second KEN box in Mad3 is not required for APC/C inhibition by Mad3-Slp1 (Cdc20), we speculate that Mad3 may sequester Slp1 (Cdc20) from the APC/C, resulting in its delayed activation. Clearly, considerable further work will be needed to elucidate the relevant phosphorylation sites of the Mad3-Slp1 (Cdc20) complex and its mechanism of action.

It is now well accepted that the presence of the Mad1-Mad2 complex at unattached kinetochores, rather than spindle damage per se, generates the MCC. The spindle assembly

checkpoint monitors kinetochore attachment rather than spindle assembly and is, therefore, poorly named. More recent studies have suggested that the Mad1-Mad2 complex at nuclear pore complexes can also generate an inhibitory signal that delays anaphase onset, although the nature of the resulting APC/C inhibitor is not known (Rodriguez-Bravo et al., 2014; Schweizer et al., 2013). In this study, we show that a distinct complex, Mad3-Cdc20, which neither contains Mad2 nor requires Mad2 for its formation, can also inhibit anaphase onset. Because pre-anaphase spindles frequently collapse in the absence of Klp9, one possibility, which deserves further study, is that the Sgo2-dependent pathway described here, in fact, monitors spindle assembly. Work in animal cells has shown that, when expression of MKLP2 is suppressed by RNAi, Aurora B kinase remains bound to the centromere and that Bub1 and BubR1, but not Mad1 and Mad2, continue to associate with kinetochores during anaphase B (Vázquez-Novelle and Petronczki, 2010). However, SAC silencing, APC/C activation, and cyclin B degradation are unaltered in this situation. One possibility is that partial knockdown of MKLP2 by RNAi is not sufficient to maintain this alternative APC/C inhibitory pathway and that this effect can only be revealed in MKLP2$^{-/-}$ null cells. Alternatively, the contribution of BBC to inhibition of the APC/C may be masked by the action of the MCC (Mad2-BubR1-Bub3-Cdc20) generated from unattached kinetochores and nuclear pores. Further experiments will be needed to distinguish between these and other intriguing possibilities.

EXPERIMENTAL PROCEDURES

Cell Culture and Strain Construction

Media and growth and maintenance of strains were as described previously (Moreno et al., 1991). All experiments were performed at 30°C unless otherwise stated. Genetic constructions are detailed in the Supplemental Experimental Procedures together with a full list of strains (Table S1) and oligonucleotides (Table S2).

Immunoprecipitation and Western Blotting

Standard procedures were used throughout (Supplemental Experimental Procedures).

Fluorescence Microscopy

Fluorescence imaging of cells expressing GFP, CFP, or TdTomato-tagged proteins was performed on a Nikon TE-2000 inverted microscope with a 100 × 1.49 numerical aperture (N.A.) objective lens equipped with a Photometrics Coolsnap-HQ2 liquid cooled charge-coupled device (CCD) camera. Images were collected and analyzed using MetaMorph (version 7.5.2.0 MAG, Biosystems Software). An exposure time of 1 s was used for GFP, CFP, and TdTomato and 0.25 s for DAPI. Maximum intensity projections were made, followed by intensity adjustments and conversion to 24-bit tagged image file format (TIFF) images for presentation.

Live-Cell Analysis of Mitotic Progression

Live-cell analysis was performed in an imaging chamber (CoverWell PCI-2.5, Grace Bio-Labs) filled with 1 mL of 1% agarose in minimal medium and sealed with a 22 × 22 mm glass coverslip. Stacks of six z sections (0.6 μm apart) were taken at each time point. The position of the spindle poles, kinetochores, or centromeres was determined using MetaMorph software.

Measurement of the Pre-anaphase Mitotic Index

Mid-log phase *ndc80-GFP cdc11-cfp*, *dad1-GFP sid4-TdTomato*, or *cdc13-GFP* strains were fixed in 3.7% formaldehyde for 10 min and mounted in medium containing DAPI to label DNA. Stacks of 18 z sections (0.2 μm apart) were taken, and the percentage of cells with Cdc13-GFP on the spindle pole bodies (SPBs) and mitotic spindle or the percentage of cells with kinetochores between separated SPBs prior to anaphase was determined. For each experiment, at least 400 cells were counted, and each experiment was conducted at least three times.

Mini-chromosome Loss Assay

Loss of the mini-chromosome was assayed as described previously (Niwa et al., 1989). Cells were grown to mid-log phase in Edinburgh minimal medium lacking adenine and then plated onto yeast extract agar containing no additional adenine for 3 days at 30°C. Half-sectored or greater than half-sectored pink colonies were scored.

SUPPLEMENTAL INFORMATION

Supplemental Information includes Supplemental Experimental Procedures, five figures, and two tables and can be found with this article online at http://dx.doi.org/10.1016/j.celrep.2017.01.032.

AUTHOR CONTRIBUTIONS

J.C.M. performed all experiments except those shown in Figures 1C and 1I (left panels, G.J.B.) and Figure S5C (A.M.S. and K.G.H.). T.C.L., J.C.M., and J.B.A.M. constructed the strains. L.J.M. and M.d.M.M.S. advised on imaging and biochemistry, respectively. J.C.M. prepared the figures, and J.B.A.M. wrote the manuscript with contributions from J.C.M.

ACKNOWLEDGMENTS

We are grateful to F. Chang, D. McCollum, Y. Watanabe, J. Javerzat, P. Russell, K. Gould, V. Simanis, M. Yanagida, S. Hauf, P. Tran, and I. Hagan for supplying strains and K. Gull for the TAT1 antibody. This work was supported by a Global Research Fellowship from the Warwick Institute of Advanced Study (to J.C.M.), program grant MR/K001000/1 from the Medical Research Council UK (to J.B.M.), and program grant 083610 from the Wellcome Trust (to K.G.H.).

Received: November 14, 2016
Revised: December 8, 2016
Accepted: January 15, 2017
Published: February 7, 2017

REFERENCES

Blanco, M.A., Sánchez-Díaz, A., de Prada, J.M., and Moreno, S. (2000). APC(ste9/srw1) promotes degradation of mitotic cyclins in G(1) and is inhibited by cdc2 phosphorylation. EMBO J. *19*, 3945–3955.

Bratman, S.V., and Chang, F. (2007). Stabilization of overlapping microtubules by fission yeast CLASP. Dev. Cell *13*, 812–827.

Browning, H., Hackney, D.D., and Nurse, P. (2003). Targeted movement of cell end factors in fission yeast. Nat. Cell Biol. *5*, 812–818.

Carmena, M., Wheelock, M., Funabiki, H., and Earnshaw, W.C. (2012). The chromosomal passenger complex (CPC): from easy rider to the godfather of mitosis. Nat. Rev. Mol. Cell Biol. *13*, 789–803.

Chao, W.C., Kulkarni, K., Zhang, Z., Kong, E.H., and Barford, D. (2012). Structure of the mitotic checkpoint complex. Nature *484*, 208–213.

Chen, J.S., Broadus, M.R., McLean, J.R., Feoktistova, A., Ren, L., and Gould, K.L. (2013). Comprehensive proteomics analysis reveals new substrates and regulators of the fission yeast clp1/cdc14 phosphatase. Mol. Cell. Proteomics *12*, 1074–1086.

Choi, S.H., and McCollum, D. (2012). A role for metaphase spindle elongation forces in correction of merotelic kinetochore attachments. Curr. Biol. *22*, 225–230.

De Antoni, A., Pearson, C.G., Cimini, D., Canman, J.C., Sala, V., Nezi, L., Mapelli, M., Sironi, L., Faretta, M., Salmon, E.D., and Musacchio, A. (2005). The

Mad1/Mad2 complex as a template for Mad2 activation in the spindle assembly checkpoint. Curr. Biol. *15*, 214–225.

Dischinger, S., Krapp, A., Xie, L., Paulson, J.R., and Simanis, V. (2008). Chemical genetic analysis of the regulatory role of Cdc2p in the S. pombe septation initiation network. J. Cell Sci. *121*, 843–853.

Esteban, V., Blanco, M., Cueille, N., Simanis, V., Moreno, S., and Bueno, A. (2004). A role for the Cdc14-family phosphatase Flp1p at the end of the cell cycle in controlling the rapid degradation of the mitotic inducer Cdc25p in fission yeast. J. Cell Sci. *117*, 2461–2468.

Foster, S.A., and Morgan, D.O. (2012). The APC/C subunit Mnd2/Apc15 promotes Cdc20 autoubiquitination and spindle assembly checkpoint inactivation. Mol. Cell *47*, 921–932.

Fu, C., Ward, J.J., Loiodice, I., Velve-Casquillas, G., Nedelec, F.J., and Tran, P.T. (2009). Phospho-regulated interaction between kinesin-6 Klp9p and microtubule bundler Ase1p promotes spindle elongation. Dev. Cell *17*, 257–267.

Gruneberg, U., Neef, R., Honda, R., Nigg, E.A., and Barr, F.A. (2004). Relocation of Aurora B from centromeres to the central spindle at the metaphase to anaphase transition requires MKlp2. J. Cell Biol. *166*, 167–172.

Han, J.S., Holland, A.J., Fachinetti, D., Kulukian, A., Cetin, B., and Cleveland, D.W. (2013). Catalytic assembly of the mitotic checkpoint inhibitor BubR1-Cdc20 by a Mad2-induced functional switch in Cdc20. Mol. Cell *51*, 92–104.

Heinrich, S., Sewart, K., Windecker, H., Langegger, M., Schmidt, N., Hustedt, N., and Hauf, S. (2014). Mad1 contribution to spindle assembly checkpoint signalling goes beyond presenting Mad2 at kinetochores. EMBO Rep. *15*, 291–298.

Hümmer, S., and Mayer, T.U. (2009). Cdk1 negatively regulates midzone localization of the mitotic kinesin Mklp2 and the chromosomal passenger complex. Curr. Biol. *19*, 607–612.

Izawa, D., and Pines, J. (2015). The mitotic checkpoint complex binds a second CDC20 to inhibit active APC/C. Nature *517*, 631–634.

Kabeche, L., and Compton, D.A. (2013). Cyclin A regulates kinetochore microtubules to promote faithful chromosome segregation. Nature *502*, 110–113.

Kaisari, S., Sitry-Shevah, D., Miniowitz-Shemtov, S., and Hershko, A. (2016). Intermediates in the assembly of mitotic checkpoint complexes and their role in the regulation of the anaphase-promoting complex. Proc. Natl. Acad. Sci. USA *113*, 966–971.

Kawashima, S.A., Tsukahara, T., Langegger, M., Hauf, S., Kitajima, T.S., and Watanabe, Y. (2007). Shugoshin enables tension-generating attachment of kinetochores by loading Aurora to centromeres. Genes Dev. *21*, 420–435.

Kawashima, S.A., Yamagishi, Y., Honda, T., Ishiguro, K., and Watanabe, Y. (2010). Phosphorylation of H2A by Bub1 prevents chromosomal instability through localizing shugoshin. Science *327*, 172–177.

King, E.M., Rachidi, N., Morrice, N., Hardwick, K.G., and Stark, M.J. (2007). Ipl1p-dependent phosphorylation of Mad3p is required for the spindle checkpoint response to lack of tension at kinetochores. Genes Dev. *21*, 1163–1168.

Kitagawa, M., Fung, S.Y., Hameed, U.F., Goto, H., Inagaki, M., and Lee, S.H. (2014). Cdk1 coordinates timely activation of MKlp2 kinesin with relocation of the chromosome passenger complex for cytokinesis. Cell Rep. *7*, 166–179.

Kulukian, A., Han, J.S., and Cleveland, D.W. (2009). Unattached kinetochores catalyze production of an anaphase inhibitor that requires a Mad2 template to prime Cdc20 for BubR1 binding. Dev. Cell *16*, 105–117.

Lara-Gonzalez, P., Scott, M.I., Diez, M., Sen, O., and Taylor, S.S. (2011). BubR1 blocks substrate recruitment to the APC/C in a KEN-box-dependent manner. J. Cell Sci. *124*, 4332–4345.

Lara-Gonzalez, P., Westhorpe, F.G., and Taylor, S.S. (2012). The spindle assembly checkpoint. Curr. Biol. *22*, R966–R980.

Lee, S.H., McCormick, F., and Saya, H. (2010). Mad2 inhibits the mitotic kinesin MKlp2. J. Cell Biol. *191*, 1069–1077.

Mansfeld, J., Collin, P., Collins, M.O., Choudhary, J.S., and Pines, J. (2011). APC15 drives the turnover of MCC-CDC20 to make the spindle assembly checkpoint responsive to kinetochore attachment. Nat. Cell Biol. *13*, 1234–1243.

Mapelli, M., Filipp, F.V., Rancati, G., Massimiliano, L., Nezi, L., Stier, G., Hagan, R.S., Confalonieri, S., Piatti, S., Sattler, M., and Musacchio, A. (2006). Determinants of conformational dimerization of Mad2 and its inhibition by p31comet. EMBO J. *25*, 1273–1284.

Meadows, J.C., Shepperd, L.A., Vanoosthuyse, V., Lancaster, T.C., Sochaj, A.M., Buttrick, G.J., Hardwick, K.G., and Millar, J.B. (2011). Spindle checkpoint silencing requires association of PP1 to both Spc7 and kinesin-8 motors. Dev. Cell *20*, 739–750.

Miniowitz-Shemtov, S., Eytan, E., Ganoth, D., Sitry-Shevah, D., Dumin, E., and Hershko, A. (2012). Role of phosphorylation of Cdc20 in p31(comet)-stimulated disassembly of the mitotic checkpoint complex. Proc. Natl. Acad. Sci. USA *109*, 8056–8060.

Mirchenko, L., and Uhlmann, F. (2010). Sli15(INCENP) dephosphorylation prevents mitotic checkpoint reengagement due to loss of tension at anaphase onset. Curr. Biol. *20*, 1396–1401.

Mora-Santos, M.D., Hervas-Aguilar, A., Sewart, K., Lancaster, T.C., Meadows, J.C., and Millar, J.B. (2016). Bub3-Bub1 Binding to Spc7/KNL1 Toggles the Spindle Checkpoint Switch by Licensing the Interaction of Bub1 with Mad1-Mad2. Curr. Biol. *26*, 2642–2650.

Moreno, S., Klar, A., and Nurse, P. (1991). Molecular genetic analysis of fission yeast Schizosaccharomyces pombe. Methods Enzymol. *194*, 795–823.

Nilsson, J., Yekezare, M., Minshull, J., and Pines, J. (2008). The APC/C maintains the spindle assembly checkpoint by targeting Cdc20 for destruction. Nat. Cell Biol. *10*, 1411–1420.

Niwa, O., Matsumoto, T., Chikashige, Y., and Yanagida, M. (1989). Characterization of Schizosaccharomyces pombe minichromosome deletion derivatives and a functional allocation of their centromere. EMBO J. *8*, 3045–3052.

Rattani, A., Vinod, P.K., Godwin, J., Tachibana-Konwalski, K., Wolna, M., Malumbres, M., Novák, B., and Nasmyth, K. (2014). Dependency of the spindle assembly checkpoint on Cdk1 renders the anaphase transition irreversible. Curr. Biol. *24*, 630–637.

Rodriguez-Bravo, V., Maciejowski, J., Corona, J., Buch, H.K., Collin, P., Kanemaki, M.T., Shah, J.V., and Jallepalli, P.V. (2014). Nuclear pores protect genome integrity by assembling a premitotic and Mad1-dependent anaphase inhibitor. Cell *156*, 1017–1031.

Sanchez-Perez, I., Renwick, S.J., Crawley, K., Karig, I., Buck, V., Meadows, J.C., Franco-Sanchez, A., Fleig, U., Toda, T., and Millar, J.B. (2005). The DASH complex and Klp5/Klp6 kinesin coordinate bipolar chromosome attachment in fission yeast. EMBO J. *24*, 2931–2943.

Schweizer, N., Ferrás, C., Kern, D.M., Logarinho, E., Cheeseman, I.M., and Maiato, H. (2013). Spindle assembly checkpoint robustness requires Tpr-mediated regulation of Mad1/Mad2 proteostasis. J. Cell Biol. *203*, 883–893.

Sczaniecka, M., Feoktistova, A., May, K.M., Chen, J.S., Blyth, J., Gould, K.L., and Hardwick, K.G. (2008). The spindle checkpoint functions of Mad3 and Mad2 depend on a Mad3 KEN box-mediated interaction with Cdc20-anaphase-promoting complex (APC/C). J. Biol. Chem. *283*, 23039–23047.

Tang, Z., Bharadwaj, R., Li, B., and Yu, H. (2001). Mad2-Independent inhibition of APCCdc20 by the mitotic checkpoint protein BubR1. Dev. Cell *1*, 227–237.

Teichner, A., Eytan, E., Sitry-Shevah, D., Miniowitz-Shemtov, S., Dumin, E., Gromis, J., and Hershko, A. (2011). p31comet Promotes disassembly of the mitotic checkpoint complex in an ATP-dependent process. Proc. Natl. Acad. Sci. USA *108*, 3187–3192.

Tsukahara, T., Tanno, Y., and Watanabe, Y. (2010). Phosphorylation of the CPC by Cdk1 promotes chromosome bi-orientation. Nature *467*, 719–723.

Uzunova, K., Dye, B.T., Schutz, H., Ladurner, R., Petzold, G., Toyoda, Y., Jarvis, M.A., Brown, N.G., Poser, I., Novatchkova, M., et al. (2012). APC15 mediates CDC20 autoubiquitylation by APC/C(MCC) and disassembly of the mitotic checkpoint complex. Nat. Struct. Mol. Biol. *19*, 1116–1123.

Vanoosthuyse, V., Prykhozhij, S., and Hardwick, K.G. (2007). Shugoshin 2 regulates localization of the chromosomal passenger proteins in fission yeast mitosis. Mol. Biol. Cell *18*, 1657–1669.

Vázquez-Novelle, M.D., and Petronczki, M. (2010). Relocation of the chromosomal passenger complex prevents mitotic checkpoint engagement at anaphase. Curr. Biol. *20*, 1402–1407.

Vázquez-Novelle, M.D., Sansregret, L., Dick, A.E., Smith, C.A., McAinsh, A.D., Gerlich, D.W., and Petronczki, M. (2014). Cdk1 inactivation terminates mitotic checkpoint surveillance and stabilizes kinetochore attachments in anaphase. Curr. Biol. *24*, 638–645.

Westhorpe, F.G., Tighe, A., Lara-Gonzalez, P., and Taylor, S.S. (2011). p31comet-mediated extraction of Mad2 from the MCC promotes efficient mitotic exit. J. Cell Sci. *124*, 3905–3916.

Wolfe, B.A., and Gould, K.L. (2004). Fission yeast Clp1p phosphatase affects G2/M transition and mitotic exit through Cdc25p inactivation. EMBO J. *23*, 919–929.

Wolfe, B.A., McDonald, W.H., Yates, J.R., 3rd, and Gould, K.L. (2006). Phospho-regulation of the Cdc14/Clp1 phosphatase delays late mitotic events in S. pombe. Dev. Cell *11*, 423–430.

Yamagishi, Y., Honda, T., Tanno, Y., and Watanabe, Y. (2010). Two histone marks establish the inner centromere and chromosome bi-orientation. Science *330*, 239–243.

Zich, J., Sochaj, A.M., Syred, H.M., Milne, L., Cook, A.G., Ohkura, H., Rappsilber, J., and Hardwick, K.G. (2012). Kinase activity of fission yeast Mph1 is required for Mad2 and Mad3 to stably bind the anaphase promoting complex. Curr. Biol. *22*, 296–301.

Zich, J., May, K., Paraskevopoulos, K., Sen, O., Syred, H.M., van der Sar, S., Patel, H., Moresco, J.J., Sarkeshik, A., Yates, J.R., 3rd., et al. (2016). Mps1Mph1 Kinase Phosphorylates Mad3 to Inhibit Cdc20Slp1-APC/C and Maintain Spindle Checkpoint Arrests. PLoS Genet. *12*, e1005834.

Cell Reports, Volume *18*

Supplemental Information

Identification of a Sgo2-Dependent but Mad2-Independent Pathway Controlling Anaphase Onset in Fission Yeast

John C. Meadows, Theresa C. Lancaster, Graham J. Buttrick, Alicja M. Sochaj, Liam J. Messin, Maria del Mar Mora-Santos, Kevin G. Hardwick, and Jonathan B.A. Millar

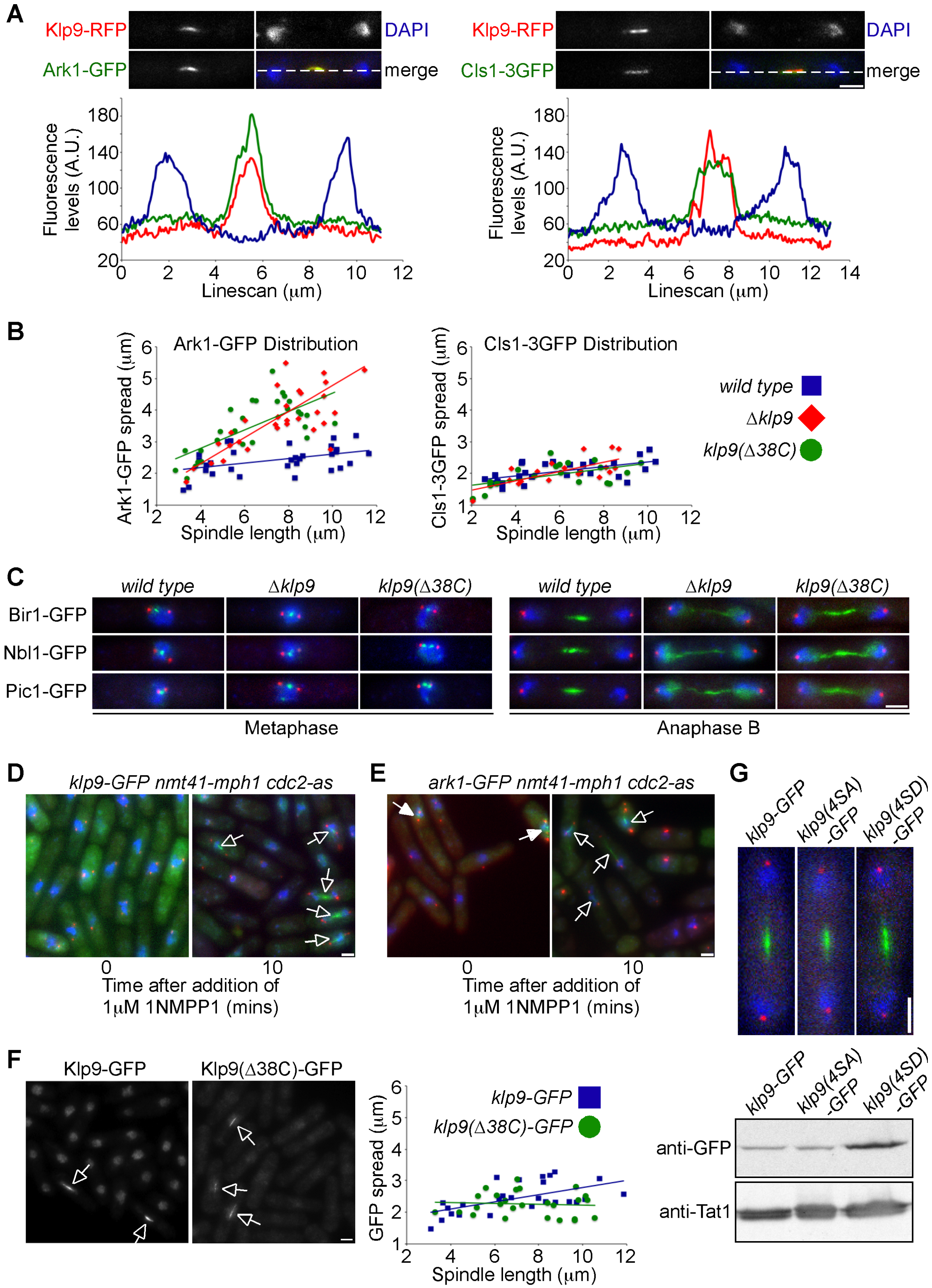

Figure S1, relating to Figure 1

Figure S1, relating to Figure 1. CPC component re-localisation requires Klp9 C-terminus and is promoted by Cdk1 inactivity

(A) Klp9 co-localises with Ark1 and Cls1 on the spindle midzone in anaphase B. Representative cells (top row panels) expressing Klp9-RFP (red) and either Ark1-GFP or Cls1-3GFP (green) with chromatin stained with DAPI (blue). All bars, 2μm. The dashed white line represents a 1 pixel wide linescan with relative fluorescence levels graphed directly below.
(B) Scatter plots to quantify Ark1-GFP and Cls1-3GFP distribution against pole-to-pole spindle length in the presence of either full length ("*wild type*"), truncated ("*klp9(Δ38C)*") or absent ("Δ*klp9*") Klp9 as shown in Figures 1B & 1G.
(C) Re-localisation of CPC components Bir1, Nbl1 & Pic1 is disrupted in the absence of the C-terminus of Klp9. Representative images of cells expressing either Bir1-GFP, Nbl1-GFP or Pic1-GFP (all green), Sid4-TdTomato (SPBs, red) and chromatin stained with DAPI (blue) in metaphase and anaphase B in either the absence of Klp9 ("Δ*klp9*"), or presence of C-terminally truncated ("*klp9(Δ38C)*") or full length Klp9 ("*wild type*").
(D) Inactivation of Cdk1 promotes localisation of Klp9 to the spindle midzone. Cells expressing Klp9-GFP (green), Sid4-TdTomato (SPBs, red), Cdc2-as (analogue-sensitive Cdc2) and a thiamine repressible promoter Mph1 construct (*nmt41-mph1*) were grown to mid-log phase in in the presence of thiamine and then transferred to medium lacking thiamine to overexpress Mph1 for 16 hours. Note lack of Klp9-GFP signal at time "0" (left panel). 10 minutes after inactivation of Cdc2 by addition of 1μM 1NMPP1, Klp9-GFP can be seen on spindles (arrows, right panel).
(E) Inactivation of Cdk1 promotes relocalisation of Ark1 to the spindle midzone. Experiment as for (D) but using cells expressing Ark1-GFP in place of Klp9-GFP. Metaphase-arrested cells (left panel, "0") have Ark1-GFP on centromeres (closed arrow-heads). Cells imaged 10 minutes after the addition of 1¨M of 1NMPP1 to inhibit Cdc2 phosphorylation (right panel, "10") show that Ark1-GFP now localises to spindles (open arrow-heads).
(F) C-terminally truncated Klp9 localises to the spindle midzone in anaphase B. Cells expressing Klp9-GFP or Klp9(Δ38C)-GFP (left panels) were grown to mid-log phase and fixed. Arrows indicate mitotic cells with GFP signals on anaphase cells. Graph shows quantification of Klp9-GFP versus Klp9(Δ38C)-GFP signals as a function of spindle length.
(G) Phospho-mimetic and non-phosphorylatable Klp9-GFP localisation and expression levels. Cells expressing Klp9-GFP, Klp9(4SA)-GFP and Klp9(4SD)-GFP (top panel) were grown to mid-log phase and fixed. Western blot (bottom panel) of mid-log phase *klp9-GFP*, *klp9(4SA)-GFP* and *klp9(4SD)-GFP* cell extracts probed with anti-GFP antibody. Levels of tubulin (anti-Tat1) are used as a loading control.

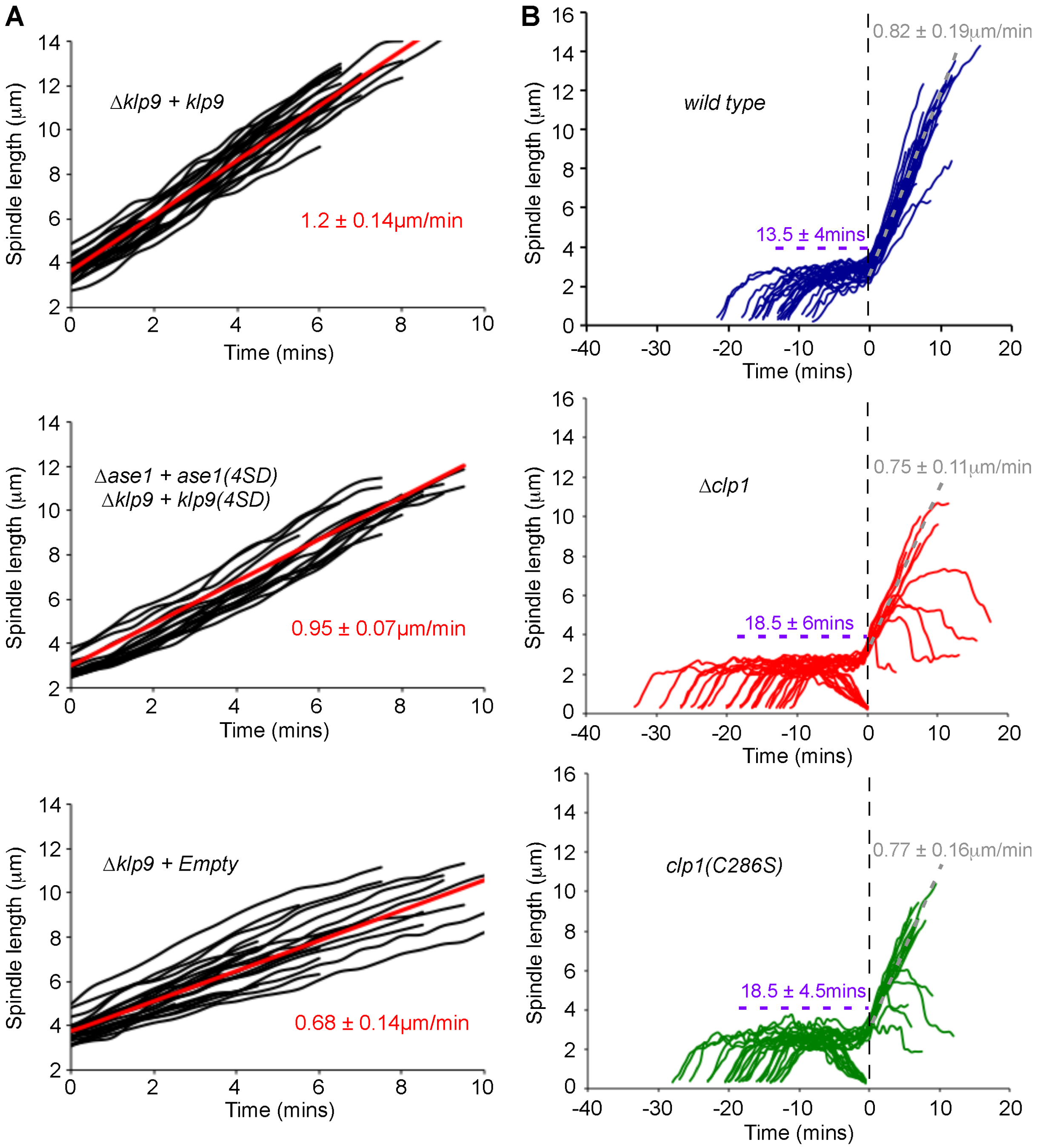

Figure S2, relating to Figure 1

Figure S2, relating to Figure 1. Neither dephosphorylation of the Klp9 C-terminus nor Cdc14-like phosphatase activity influence Anaphase B spindle elongation rate

(A) C-terminal phosphomimetic *ase1* and *klp9* alleles do not alter the rate of anaphase B spindle elongation. Log phase *sid4-TdTomato* cells deleted for *klp9* and with either *klp9 (Δklp9 + klp9*, top panel, n= 25) or empty plasmid (Δ*klp9* + *Empty*, bottom panel, n= 23) integrated or the same with *ase1* also deleted and expressing phosphomimetic *ase1* and *klp9* constructs (Δ*ase1* + *ase1(4SD)* Δ*klp9* + *klp9(4SD)*, middle panel, n= 17) were imaged by fluorescence microscopy at 30°C. Spindle length in individual anaphase B cells was calculated at 30 second intervals. Red lines indicate mean spindle elongation rates, which are quantified ± standard deviation.
(B) Neither the presence nor the activity of Cdc14-like phosphatase, Clp1, alter the rate of spindle elongation in anaphase B. Log phase *ndc80-GFP cdc11-CFP* cells (*wild type*, top panel, n = 29) or the same lacking Clp1 (Δ*clp1*, middle panel, n = 25) or expressing Clp1(C286S) (*clp1(C286S)*, bottom panel, n = 25) were imaged by fluorescence microscopy at 30°C. Spindle length in individual mitotic cells was calculated at 30 second intervals. The completion of anaphase A was taken as T = 0 for each movie. Spindle collapses are those traces in which spindle length reduces to zero. Average time in prometaphase & metaphase and anaphase B spindle elongation rate are shown ± standard deviation.

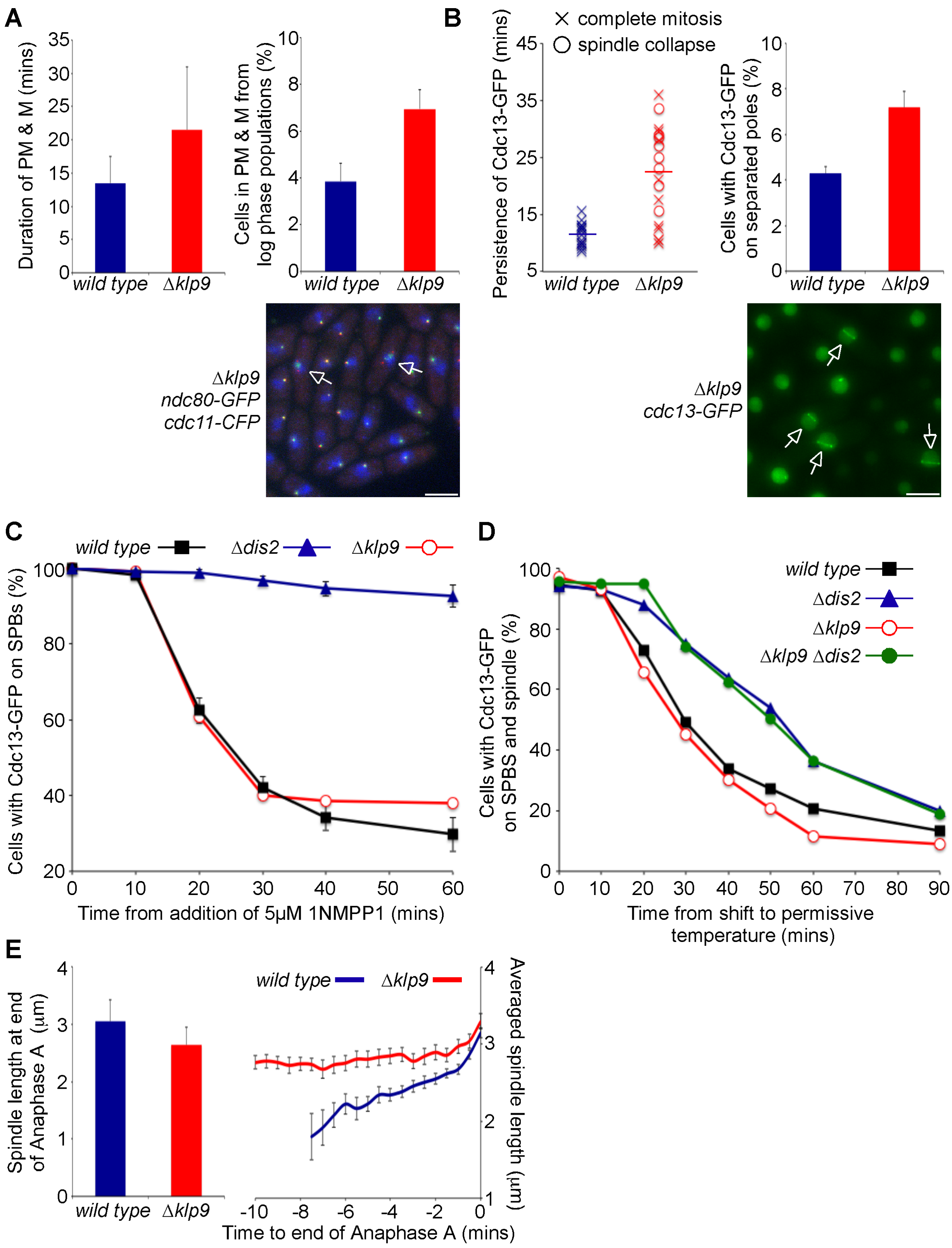

Figure S3, relating to Figure 2

Figure S3, relating to Figure 2. Klp9 is required for timely Cdc13 destruction due to prolonged SAC activation rather than defective silencing

(A) Comparison of the duration of prometaphase and metaphase (PM & M) from live cell analysis in Figure 2A with the proportion of PM & M cells in fixed images of mid-log phase cells either in the presence (*wild type*) or absence (Δ*klp9*) of Klp9. Error bars show standard deviation. Representative image (bottom panel) with PM & M cells highlighted with arrowheads. Spindle poles fluorescently-tagged with Cdc11-CFP (red) and kinetochores with Ndc80-GFP (green). All bars, 5µm.
(B) Cdc13 localisation persists in cells deleted for Klp9. Cells with and without Klp9 and expressing Cdc13-GFP were grown to mid-log phase at 30°C before conducting either live cell analysis (left panel) to determine the duration of GFP signal in individual mitotic cells or being fixed (right panel) and scored for GFP signal on separated poles. Horizontal lines show mean values (left panel) and error bars represent standard deviation (right panel). Representative image (bottom panel) with cells that localise Cdc13-GFP (green) on separated poles highlighted with arrowheads.
(C) Klp9 is not required for microtubule-independent spindle checkpoint silencing. Cells of the indicated genotypes expressing Cdc13-GFP (to monitor mitotic progression), Ark1-as3 (analogue-sensitive Aurora B to inactivate the spindle checkpoint) and Nda3-KM311 (cold-sensitive ß-tubulin mutant to arrest cells in a prometaphase-like state) were grown to mid-log phase at 30°C before being arrested for 6 hours at 18°C. 1NMPP1 was then added and samples collected at the indicated time points and assayed for the presence of Cdc13-GFP on poles.
(D) Klp9 does not influence the timing of anaphase onset following release from a spindle checkpoint arrest. Cdc13-GFP expressing cells containing the *nda3-KM311* allele were grown to mid-log phase at 30°C before being arrested in prometaphase-like state by shifting temperature to 18°C for 6 hours. Cells were then released at 30°C and the proportion of cells with Cdc13-GFP on poles and spindles assayed at the indicated time points.
(E) Spindle length at the end of anaphase A is not altered in the absence of Klp9. Left panel shows data extracted from time 0 in Figure 2A and the right panel shows data from the same cells that are analysed in Figure 2C.

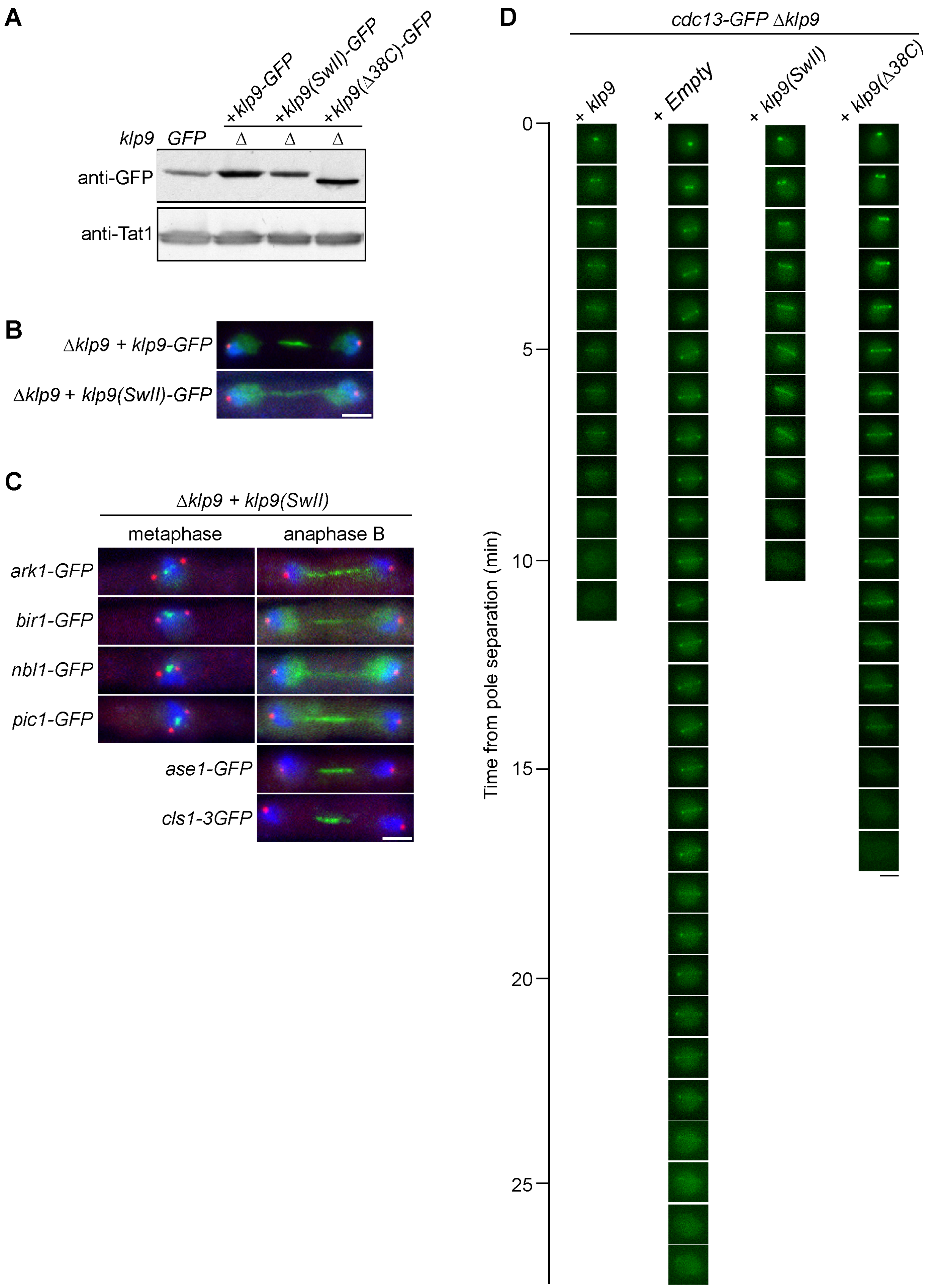

Figure S4, relating to Figure 3

Figure S4, relating to Figure 3. Klp9(SwII) characterisation

(A) Expression levels of *klp9:leu1* constructs. Mid-log phase *klp9-GFP,* Δ*klp9 + klp9-GFP,* Δ*klp9 + klp9(SwII)-GFP* and *klp9 + klp9(*Δ*38C)-GFP* cell extracts were probed with anti-GFP antibody. Levels of tubulin (anti-Tat1) are used as a loading control.
(B) Klp9-SwII does not localise to the spindle midzone. Δ*klp9* cells expressing either Klp9-GFP or Klp9(SwII)-GFP (green) form the *leu1* locus and Sid4-TdTomato (red) were grown to mid-log phase at 30°C and fixed. All bars, 2μm.
(C) CPC component relocalisation to the spindle midzone is disrupted in the absence of Klp9 motor activity. Δ*klp9 + klp9(SwII) sid4-TdTomato* cells expressing either Ark1-GFP, Bir1-GFP, Nbl1-GFP, Pic1-GFP, Ase1-GFP or Cls1-3GFP (green) were grown to mid-log phase at 30°C and fixed.
(D) Timely Cyclin B destruction requires the C-terminus of Klp9 but not its motor activity. Representative movies from the data quantified in Figure 3D.

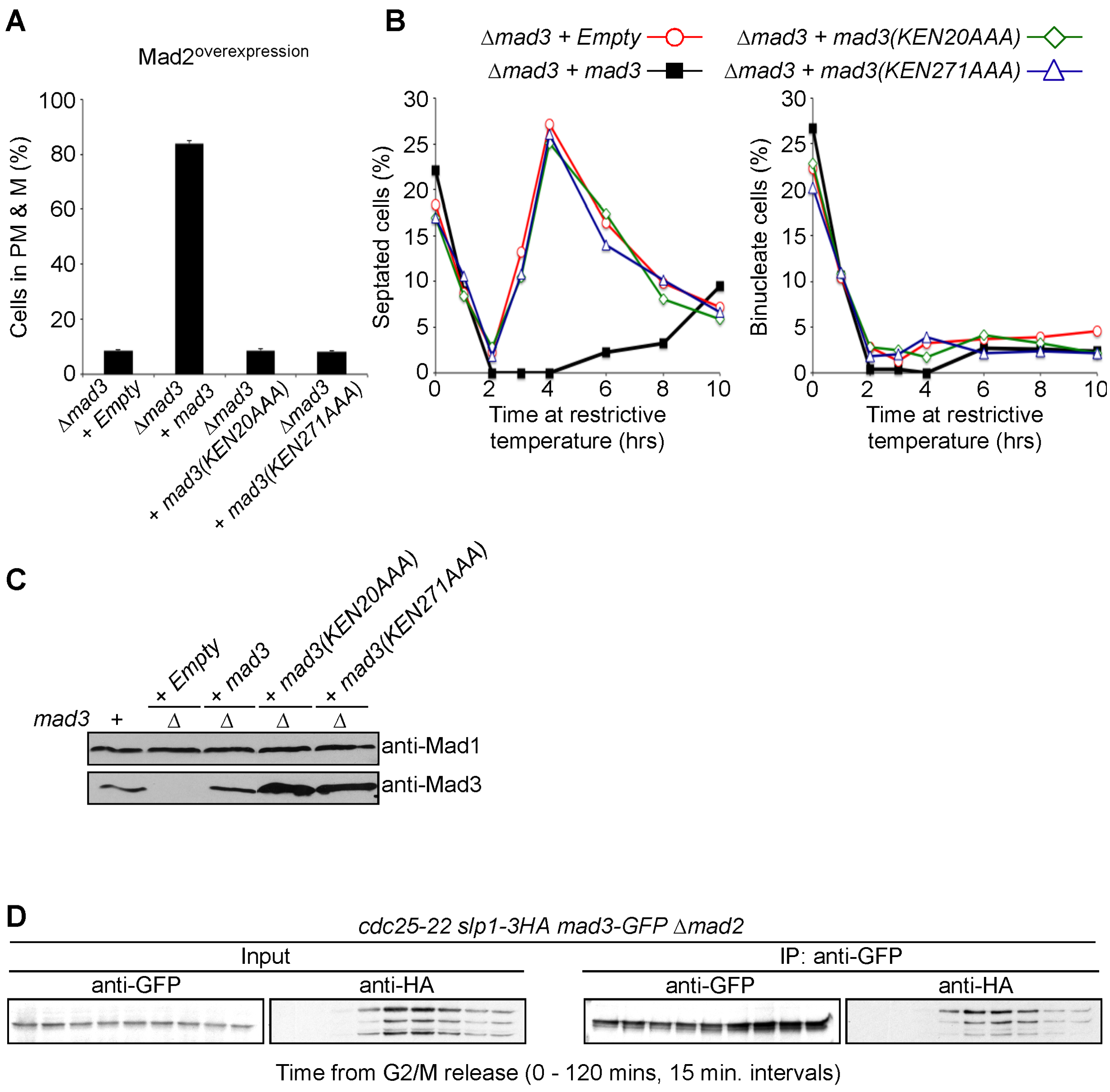

Figure S5, relating to Figure 5

Figure S5, relating to Figure 5. SAC dependency controls

(A) Mad3-KEN20AAA and Mad3-KEN271AAA are both defective in checkpoint arrest in response to over-expression of Mad2. *dad1-GFP sid4-TdTomato* cells deleted for Mad3 and expressing either empty plasmid (*Empty*), *mad3*, *mad3-KEN20AAA* or *mad3-KEN271AAA* were grown to mid-log phase at 30°C in media containing thiamine. They were then washed and resuspended in media lacking thiamine to allow overexpression of Mad2 under the control of a thiamine repressible promoter. After 16 hours cells were fixed and the proportion in Prometaphase & Metaphase (PM & M) assayed by fluorescence microscopy.
(B) Mad3-KEN20AAA and Mad3-KEN271AAA both fail to mount a checkpoint response to *nda3-KM311*-mediated checkpoint activation. Mid-log phase *nda3-KM311* cells with the genotypes detailed were shifted from 30°C to 18°C at time zero. Cells were sampled at the times indicated and stained with both calcofluor to assay the proportion of septated cells (left panel) and DAPI to stain chromatin and score the proportion of binucleate cells (right panel).
(C) Mad3 KEN box mutant proteins are present at wild type Mad3 levels. Lysates were prepared from log phase strains expressing either endogenous *mad3* or the various *mad3* constructs integrated at the *leu1* locus. Levels of Mad1 and Mad3 were assessed following western blotting.
(D) Mad3 and Slp1 interact in the absence of Mad2. *cdc25-22 slp1-3HA mad3-GFP* Δ*mad2* cells were arrested for 4 hours at 35.5˚C to synchronise at the G2/M transition. Following release at 25˚C, extracts were prepared every 15 minutes. Association between Slp1-3HA and Mad3-GFP was assessed by immunoprecipitation and western blot.

Table S1

List of fission yeast strains used in this study.

All strains are *ura4-D18 leu1-32*. *ade6* allele is unknown unless stated.

Table S2

List of oligonucleotides used in this study.

Bir1 tag.F	GAGTTTGAAGAAGCCTGTGAAGAAAAAATAGAATGGTT ACTGGAAGAAGGTAAGCGGGCGGAAGAATACATACAA AACTTACGGATCCCCGGGTTAATTAA
Bir1 tag.Rev	CACATTACTTTAAAATGTAGCCCGCCAACATTTTCGCTT AAAGCGAGACGGTCCATAAATACAGAAACACAATATG TAAAAGAATTCGAGCTCGTTTAAAC
Klp9 del.F	AGCAACTGTCTTCCAACACTTCCATCATTCTTGTACACA AAGGATTGCTTTTAAGTCCCTTTAAACTCTGAAATTGAC CACGGATCCCCGGGTTAATTAA
Klp9 tag.F	TAAAACCATTAAGCCCCTCTCGACGCCCTCCGTTGACTT CTTTGTATAGTGGGACAACTGATATCGATATTAATGAAT TGCGGATCCCCGGGTTAATTAA
Klp9 del/tag.R	CATGTGAAGGCAAGAGCTAAATATTACTCAAATAAGAG TTATGAACACATTTTGCCATTCTTGGTAACTTTAATGATA TAGAATTCGAGCTCGTTTAAAC
Klp9 XbaI.F	TGCTCTAGAGCAATCGCAGTC
Klp9 KpnI.R	TAAGGTACCAAATGCCTTCGTTTTAAA
Klp9 PstI.R	TTTCTGCAGTTAGGGTACGTTTTCCTCATCATC
Klp9 SwII.F	GATTTAGCCGCATCCGAGCGT
Klp9 SwII.R	CACCAAATCTATTTGACAACTTTCAAA
Klp9 S596A,S598A.F	GTACCCGCACCGGCTCCCAAAAAAAAAGTT
Klp9 S596D,S598D.F	GTACCCGATCCGGATCCCAAAAAAAAAGTT
Klp9 S596A or D.R	GTTTTCCTCATCATCACTATTTGG
Klp9 S598D,S605D.F	AGTCCGGATCCCAAAAAAAAAGTTGTAGATCCAATA
Klp9 S598D,S605D.R	GGGTACGTTTTCCTCATCATCACTATTTGG
Klp9 S605A,S611A.F	CCCAAAAAAAAAGTTGTAGCGCCAATAAAACAATTAGC GCCCTCTCGACGCCCTCCGTTG
Klp9 S605A,S611A.R	CAACGGAGGGCGTCGAGAGGGCGCTAATGGTTTTATTG GCGCTACAACTTTTTTTTTGGG
Klp9 S611D.F	CCATTAGATCCCTCTCGACGCCCT
Klp9 S611D.R	TTTTATTGGATCTACAACTTTTTTTTTGGG
Klp9(Δ38C).w	ACGATTGCGCAGTTAGAACAAATT
Klp9(Δ38C).x	GGGGATCCGTCGACCTGCAGCGTACGATTAGGGTACGT TTTCCTCATC
Klp9(Δ38C).y	GTTTAAACGAGCTCGAATTCATCGATATCCGTCGTGCAT AGTGCTTA
Klp9(Δ38C).z	AGAGCTAAATATTACTCAAATAAGAGT
Klp9(Δ38C) tag.x	GGGGATCCGTCGACCTGCAGCGTACGAGGGTACGTTTT CCTCATC
Mad3 BamHI.F	CGCGGATCCTCTTGTAAATCGTTGTTTATAAATCTTCAT
Mad3 PstI.R	TTGGATCTGCAGGGAGTTGATTTTGTTTTGGTTCAA
Mad3 NruI(mut).F	AAATCTTCGTCCCGAAACCACA
Mad3 NruI(mut).R	TGCAAGTGCAGAAGCTGAATG
Mad3 KEN20.F	GTCATTGAGCAGTCCGCGGCAGCCATAGAGCCTAGAAAA
Mad3 KEN20.R	ATCCATATGCACCCAGTTCTTGCC
Mad3 KEN271.F	GTTGATCAAAGGCGTGCAGCAGCCAACATATCGGCTACT
Mad3 KEN271.R	TGTACCAAGAGTTTGCCAGGTCCC

Supplemental Experimental Procedures

Strain construction
Δ*klp9*, *klp9-GFP*, and *bir1-13myc* were generated using a single-step PCR-based protocol (Bahler et al., 1998). *klp9(Δ38C)* was produced by two-step PCR-based gene targeting as previously described (Krawchuk and Wahls, 1999). The resulting truncation was tagged with GFP using the same method. See Table S2 for a full list of oligonucleotides used in this study.

Plasmid construction
Klp9 was cloned as a 2.8kb fragment with 500bp of promoter region and 289bp of 3' UTR and inserted into the *XbaI* and *KpnI* sites of pJK148 and clones verified by sequencing. The Δ38C mutant was generated by a truncation at residue 595 and cloned into the *XbaI* and *PstI* sites of pJK148. Serines 596, 598, 605 and 611 were mutated to either alanine or aspartic acid and the Switch II (G296A) mutant generated by Phusion site-directed mutagenesis kit (Fisher Scientific) according to manufacturer's instructions.

Mad3 was cloned as a 1.8kb fragment containing 500bp of upstream promoter sequence and 440bp of 3' UTR and cloned into the *BamHI* and *PstI* sites of pJK148. To ensure the resulting plasmid was suitable for integration, the *NruI* restriction site at Serine 40 of *mad3* was mutated from TCG to TCC using Phusion Site-directed Mutagenesis kit and clones verified by sequencing. KEN box mutations were likewise generated using the Phusion Site-directed mutagenesis kit.

Plasmids were linearised with *NruI* and integrated into *S. pombe* strains with the *leu1.32* auxotrophic marker and confirmed by PCR (Keeney and Boeke, 1994). See Table S2 for a full list of oligonucleotides used in this study.

Biochemistry
Cells were lysed in buffer containing 50 mM HEPES (pH 7.6), 75 mM KCl, 1 mM $MgCl_2$, 1 mM EGTA, 0.1% Triton X-100, 1 mM DTT, 1 mM PMSF and c0mplete EDTA-free protease inhibitor cocktail (Roche). Cleared extracts were incubated with GFP-Trap_A beads (Chromotek) for 1 hour at 4°C. Beads were washed three times in lysis buffer and boiled in sample buffer.

GFP was detected using anti-GFP sheep polyclonal antibody (1/5000) and HRP conjugated anti-sheep secondary antibody (1/20000). Myc was detected using anti-Myc rabbit polyclonal antibody (1/500: A-14, Santa Cruz) and HRP conjugated anti-rabbit secondary antibody (1/10000: GE Healthcare). HA was detected using HRP-conjugated anti-HA rat monoclonal antibody (1/500: 3F10, Roche). Mad1 and Mad3 were detected using sheep polyclonal antibodies and HRP conjugated anti-sheep secondary antibody. ECL detection was performed *via* Amersham ECL system (GE Healthcare).

Co-localisation analysis
Fluorescence levels for localisation were measured in MetaMorph using the linescan option set at one pixel width for > 10μm length. Chromatic aberrations were minimized by using a single dichroic filter for all channels.

Checkpoint-silencing assay
Performed exactly as in (Meadows et al., 2011).

Supplemental References

Bahler, J., Wu, J.Q., Longtine, M.S., Shah, N.G., McKenzie, A., 3rd, Steever, A.B., Wach, A., Philippsen, P., and Pringle, J.R. (1998). Heterologous modules for efficient and versatile PCR-based gene targeting in Schizosaccharomyces pombe. Yeast *14*, 943-951.

Keeney, J.B., and Boeke, J.D. (1994). Efficient targeted integration at leu1-32 and ura4-294 in Schizosaccharomyces pombe. Genetics *136*, 849-856.

Krawchuk, M.D., and Wahls, W.P. (1999). High-efficiency gene targeting in Schizosaccharomyces pombe using a modular, PCR-based approach with long tracts of flanking homology. Yeast *15*, 1419-1427.

Meadows, J.C., Shepperd, L.A., Vanoosthuyse, V., Lancaster, T.C., Sochaj, A.M., Buttrick, G.J., Hardwick, K.G., and Millar, J.B. (2011). Spindle checkpoint silencing requires association of PP1 to both Spc7 and kinesin-8 motors. Developmental cell *20*, 739-750.
